# Self-help guidebook improved quality of life for patients with irritable bowel syndrome

**DOI:** 10.1371/journal.pone.0181764

**Published:** 2017-07-25

**Authors:** Antonius Schneider, Stefanie Rosenberger, Johanna Bobardt, Jessica Bungartz-Catak, Oxana Atmann, Bernhard Haller, Anne Kennedy, Paul Enck

**Affiliations:** 1 Institute of General Practice, University Hospital Klinikum rechts der Isar, Technische Universität München, München, Germany; 2 Institute for Medical Statistics and Epidemiology, University Hospital Klinikum rechts der Isar, Technische Universität München, München, Germany; 3 NIHR Collaboration for Leadership in Applied Health Research (CLAHRC) Wessex, Faculty of Health Sciences, University of Southampton, Highfield Campus, Southampton, United Kingdom; 4 Department of Internal, Medicine VI: Psychosomatic Medicine and Psychotherapy, University Hospital, Tübingen, Germany; University Hospital Llandough, UNITED KINGDOM

## Abstract

**Background:**

The primary aim of our study was to evaluate the impact of a comprehensive self-help guidebook on the disease related quality of life for patients with irritable bowel syndrome (IBS). The secondary aim was to evaluate whether the guidebook is less effective in IBS patients with depression, somatization disorder or panic disorder as a psychiatric comorbidity.

**Methods:**

Prospective observational study. At baseline (t1), patients filled in the ´Functional Digestive Disorders Quality of Life´ (FDDQL) questionnaire and received the IBS guidebook together with an explanation of its content and use. Depression, anxiety and somatization were evaluated with the Patient Health Questionnaire (PHQ). Three (t2) and six months (t3) later, the questionnaire was sent by mail to the patients for follow-up evaluation. Data were analyzed with repeated measures ANOVA.

**Results:**

71 patients participated (74.6% female). 53 (74.6%) completed the final assessment at t3 after 6 months. The global FDDQL score increased from 49.3 (SD 12.7) at t1 to 64.3 (SD 16.0) at t3 (p < 0.001). There was a significant between-subjects effect on the global FDDQL score related to depression (p = 0.001), anxiety (p = 0.001) and somatization (p = 0.011). Thus, the quality of life of patients with psychosomatic comorbidity was lower at baseline, but showed a similar increase within the following six months.

**Conclusion:**

The self-help guidebook significantly improved measured quality of life for IBS patients. The use of screening questionnaires like PHQ might be valuable to identify patients with more complex problems. This might be helpful for them to intensify and adapt therapy. Further research has to evaluate if patients with psychological comorbidity are treated more effectively when they receive psychotherapy or specific medication in addition to the self-management guidebook.

## Introduction

Irritable bowel syndrome (IBS) is the most common functional gastrointestinal disorder. It is associated with a reduced quality of life and accompanied by a high level of suffering for the afflicted patients [1]. Up to now, the pathogenesis remains unclear. Evidence suggests that dysfunction in the gut-brain-axis, previous gastrointestinal infections, changes in the microbiome, visceral hypersensitivity and changes in the gastrointestinal motility may all contribute to the development of IBS [2–4]. Furthermore, studies have shown that psychosomatic comorbidity plays an important role in the development of IBS [5, 6], which was recently underlined by an epidemiological study in Germany [7].

IBS poses a therapeutic challenge due to the multifactorial nature of the disease. The chronicity of IBS symptoms leads to impaired physical function with an extensive level of suffering, increased use of secondary health care services and annual health care costs of up to 4.1 billion Euros per year in Germany [8]. Moreover, IBS related costs are significantly higher when patients are treated in secondary-care compared to primary-care settings [9,10]. Thus, treating IBS patients preferably in a primary-care setting not only complies with the guideline recommendations but also reduces the economic burden on health care resources and is therefore of vital importance.

A self-help guidebook for patients has proven to be an effective intervention in primary care settings. Robinson et al. successfully introduced the guidebook in a randomized controlled trial [11]. The original guidebook development was based on patients´ views and needs [12]. These were extracted from focus groups with IBS patients in which their key questions and preoccupations concerning their condition were explored. Patients described their experiences, coping strategies, experiences of the healthcare system, treatments and social consequences of the condition. Medical literature searches were made to find answers to identified areas of information need. The book includes direct quotes from patients describing their own experiences [12]. The use of the self-help guidebook has been shown to reduce primary care consultations by 60% and costs by 40% [11]. For the present study we adapted the second edition of the self-help guidebook by Kennedy and Robinson and adjusted it to the German health care system and the current state of research. The primary aim of our study was to evaluate the impact of a comprehensive self-help guidebook on disease related quality of life for IBS patients. The secondary aim was to evaluate whether the guidebook is less effective in IBS patients with depression, somatization disorder or panic disorder as a psychiatric comorbidity.

## Patients and methods

### Study design

The study was carried out by the Institute of General Practice, Technische Universität München and performed as a prospective observational study between June 2014 and January 2015, with assessment at baseline (t1) and follow-up after three (t2) and six (t3) months. Patients were recruited by their physician in six general practices, two private practices of gastroenterologists and via newspaper advertisements. At baseline, patients filled in a quality of life questionnaire and received the IBS guidebook together with an explanation of its content and use. At t2 and t3, the questionnaire was sent by mail to the patients for follow-up evaluation.

### Sample

Only patients fulfilling the Rome III diagnostic criteria for IBS [13] were included. Diagnosis was confirmed by the presence of abdominal pain/discomfort for a minimum of three months and with at least two of the following features: symptom relief with defecation and/or symptoms associated with a change in frequency of stools, and/or associated with a change in form (appearance) of stools, in the absence of structural or metabolic abnormalities to explain the symptoms. A diagnosis of IBS had to exclude other causes of symptoms, such as inflammatory bowel diseases, carbohydrate malabsorption, and colon cancer. Further inclusion criteria were a minimum age of 18 years, sufficient German language competency and signed written informed consent. Exclusion criteria were age under 18 years, insufficient German language competency and other gastrointestinal diseases which might explain the bowel symptoms. Patients who were recruited by newspaper advertisement were assessed by a general practitioner based at the institute (JBC). These patients were instructed to bring medical letters to guarantee the correct diagnosis of IBS and exclusion of other important differential diagnoses. The study was approved by the Ethics Committee of the Medical Faculty of the Technische Universität München.

### Self- help guidebook

The self-help guidebook evaluated in this study is an adapted and translated German version of the second edition of “Managing your Life with Irritable Bowel Syndrome” [11,12], which was adjusted to the German health care system and modified based on the German Consensus Guidelines on IBS [14]. The last update of the English version was 2009 and some information required updating due to recent evidence. We expanded the self-help guidebook and included sections on malabsorption of fructose and sorbitol, and coeliac disease. Additionally, we noted that the efficacy of acupuncture and aloe vera is due to a placebo effect. Furthermore aromatherapy and charcoal treatments are uncommon in Germany. Therefore we did not include these treatment suggestions in the German self-help guidebook. Beyond that, several herbal remedies are not available in Germany; and vice versa some German remedies were not available in UK. We adapted these accordingly and explained the lack of evidence regarding herbal medication. The German version is provided as a supplement (S1 Fig).

### Data collection

At the baseline assessment, patients completed a questionnaire to obtain sociodemographic data such as family status, level of education, professional education and occupation. Quality of life was measured using a German translation of the FDDQL (Functional Digestive Disorders Quality of Life) questionnaire [15]. 43 items are grouped for eight subscales: daily activity, disease related anxiety, diet, sleep, discomfort, health perception, coping with disease and impact of stress, with possible results ranging from 1 (poor quality of life) to 100 (very good quality of life). The FDDQL has an internal consistency of 0.94 (Crohnbach’s alpha).

Influences of depression, anxiety and somatoform disorder on quality of life after the guidebook intervention were studied. Three subscales of the German version of the Patient Health Questionnaire (PHQ) were used to assess depression, anxiety and somatisation. The depression severity score of the PHQ, the PHQ-9, comprises nine items which can be summarized [16]. The range is from 0 (no depression) to 27 (maximal depression). Superior criterion validity of the PHQ compared to other established self-report questionnaires was confirmed with respect to the diagnoses of ´major depressive disorder´ and ´other depressive disorders´ made by a standard interview in assessing psychiatric disorders [16]. The 7-item Generalized Anxiety Disorder Scale (GAD-7) was used as a practical self-report anxiety questionnaire that proved valid in primary care [17]. It has been established as a reliable and valid self-report measure for anxiety in the general population, as has the German version. GAD-7 scores range from 0 to 21, with scores of ≥ 5, ≥ 10, and ≥ 15 representing mild, moderate, and severe anxiety symptom levels, respectively. Finally, somatization was measured using the somatic symptom module of the PHQ, the PHQ-15 [18]. The PHQ-15 has high internal reliability and construct validity. The PHQ-15 inquires about 15 somatic symptoms or symptom clusters that account for more than 90% of the physical complaints reported in the outpatient setting and includes 14 of the 15 most prevalent DSM-IV somatization disorder somatic symptoms. A somatisation severity score can be derived by inclusion of two questions from the depression module. The range is from 0 (no somatisation) to 30 (severe somatisation). At least three items have to be rated as ´severe impairment´ to establish the diagnosis of a somatoform syndrome [18].

### Statistical methods

The data were analyzed using IBM SPSS Statistics for Windows, version 23 (IBM Corp., Armonk, N.Y., USA). In the description of the data, means and standard deviations are presented for quantitative measures, absolute and relative frequencies for categorical data. Two-sample t-tests were used to compare means of quantitative data between two independent groups, Pearson’s chi-squared test or Fisher’s exact test were used for comparison of the distribution of nominal data between groups. To test for systematic changes in quantitative outcomes over time, ANOVA (analysis of variance) for repeated measures was carried out, as no relevant deviation from the normal distribution was detected for changes in the considered variables. Changes in dichotomous, nominal data were analyzed using McNemar’s test.

Comparison of changes in quantitative outcomes between relevant groups (depression, anxiety, somatoform disorder at t1) were conducted using ANOVA with repeated measures with the above-mentioned variables used as between-subjects factors. Between-subjects effects were determined to describe overall group differences regarding the mean values of t1, t2 and t3, i.e. a difference in the levels of quality of life between the groups. Differences in changes over time between patient groups were analyzed by assessing the interaction between time and the distinct groups of depression, anxiety and somatization. A level of significance of p < 0.05 was used. Due to the pilot character of the study, we aimed to reach the six month follow-up for at least 50 patients. The study data are provided as supplement (S1 and S2 Tables).

## Results

A total of 71 patients fulfilled the criteria for IBS and were thus enrolled into the study. Of these, 13 patients (18.3%) were recruited by the general practitioners, 20 patients (28.2%) by the gastroenterologists and 38 patients (53.5%) by newspaper advertisements. 53 (74.6%) patients completed the final assessment at t3 after 6 months. The mean follow-up period was 6.4 months (SD 0.6). The mean age of the patients at enrollment was 47.4 (SD 18.3) years, the range was from 20.6 to 86.4 years; 74.6% (n = 53) were female (Table 1). Of all, 98.7% (n = 70) had finished primary education, 60.6% (n = 43) had graduated from high school and 57.7% (n = 41) were in full or part-time employment (Table 1).

**Table 1:** Sociodemographic gender characteristics for patient sample.

	Female n = 53 (74.6%)	Male n = 18 (25.4%)	Overall n = 71 (100.0%)	
	Mean (SD)	Mean (SD)	Mean (SD)	p-value
**Age** in years	47.23 (18.8)	48.03 (17.3)	47.4 (18.3)	0.874^a^
	**N (%)**	**N (%)**	**N (%)**	
**Marital status**				0.116^b^
**Married/ cohabiting**	30 (56.6)	15 (83.3)	45 (63.4)	
**Single**	20 (37.7)	3 (16.7)	23 (32.4)	
**Widowed/ divorced**	3 (5.7)	0 (0.0)	3 (4.2)	
**Education**				0.683^b^
**No school leaving certificate**	0 (0.0)	0 (0.0)	0 (0.0)	
**Lower secondary school certificate**	6 (11.3)	3 (16.7)	9 (12.7)	
**Secondary school certificate**	15 (28.3)	3 (16.7)	18 (25.4)	
**Educated to degree level**	31 (58.5)	12 (66.7)	43 (60.6)	
**Other**	1 (1.9)	0 (0.0)	1 (1.4)	
**Job qualification**				0.491^b^
**No job qualification**	3 (5.7)	1 (5.6)	4 (5.6)	
**Vocational school**	25 (47.2)	7 (38.9)	32 (45.1)	
**University**	20 (37.7)	10 (55.6)	30 (42.3)	
**Other**	5 (9.4)	0 (0.0)	5 (7.0)	
**Employment**				**0.024**^**b**^
**Part-time employment**	16 (30.2)	1 (5.6)	17 (23.9)	
**Full-time employment**	12 (22.6)	12 (66.7)	24 (33.8)	
**Homemaker**	3 (5.7)	0 (0.0)	3 (4.2)	
**Retired**	13 (24.5)	4 (22.2)	17 (23.9)	
**No job**	3 (5.7)	0 (0.0)	3 (4.2)	
**Other**	6 (11.3)	1 (5.6)	7 (9.9)	
**Comorbidities**	**Mean (SD)**	**Mean (SD)**	**Mean (SD)**	**p-value**
**FDDQL**	47.9 (12.7)	58.0 (12.6)	50.4 (13.3)	**0.005**^**a**^
**Depression**	9.0 (5.1)	5.7 (3.3)	8.1 (4.9)	**0.012**^**a**^
**Anxiety**	7.7 (5.9)	5.2 (3.4)	7.1 (5.5)	**0.036**^**a**^
**Somatic symptom disorder**	12.6 (5.1)	10.4 (4.2)	12.1 (5.0)	0.108^a^

p-value for gender differences (^a^two-sample-t-test, ^b^ Fisher’s exact test)

The FDDQL showed a large increase over the following six months (Table 2). The global FDDQL score increased from 49.3 (SD 12.7) at t1 to 64.3 (SD 16.0) at t3 (p < 0.001 in repeated measures ANOVA). Correspondingly, there was a significant increase of quality of life in all FDDQL subscales (p < 0.001), with the exception of the impact of stress scale (p = 0.335). Beyond that, depression, anxiety, and somatization decreased over time (p < 0.001 in repeated measures ANOVA).

**Table 2:** Quality of life and psychiatric comorbidities (FDDQL and PHQ-D) during the course of the study.

	t1	t2	t3			
FDDQL	Mean (SD)	Mean (SD)	Mean (SD)		F-value (F_crit_)	p-value
**Global** (n = 53)	49.3 (12.7)	62.0 (14.5)	64.3 (16.0)		42.165 (3.367)	**< 0.001**
**Anxiety** (n = 53)	50.4 (18.5)	68.5 (19.4)	69.5 (21.6)		29.286 (3.344)	**< 0.001**
**Coping with disease** (n = 52)	37.8 (19.7)	56.4 (23.5)	59.3 (23.6)	25.230 (3.085)		**< 0.001**
**Daily activities** (n = 53)	62.3 (18.8)	75.1 (20.5)	77.5 (24.5)	15.868 (3.724)		**< 0.001**
**Diet** (n = 52)	42.5 (25.0)	52.2 (22.0)	56.5 (23.5)	12.463 (3.423)		**< 0.001**
**Discomfort** (n = 53)	38.6 (14.7)	50.8 (19.9)	53.0 (20.6)	23.964 (3.084)		**< 0.001**
**Health perception** (n = 53)	44.0 (20.1)	50.9 (20.0)	54.1 (18.2)	8.721 (3.271)		**0.001**
**Impact of stress** (n = 51)	37.3 (26.9)	40.5 (25.6)	40.0 (25.8)	1.075 (3.342)		0.335
**Sleep** (n = 52)	70.0 (20.0)	80.0 (18.9)	80.8 (15.2)	11.962 (3.085)		**< 0.001**
**PHQ-D**						
**Depression** (n = 51)	8.2 (4.9)	5.7 (3.8)	5.8 (4.3)	12.874 (3.628)		**< 0.001**
**Anxiety** (n = 51)	7.2 (5.9)	5.5 (4.8)	5.1 (4.2)	9.789 (3.087)		**< 0.001**
**Somatic Symptoms** (n = 49)	12.4 (4.9)	10.7 (5.0)	9.9 (4.6)	10.189 (3.091)		**< 0.001**

p- and F-value for difference between t1 and t3 (ANOVA with repeated measures)

**Fig 1:**
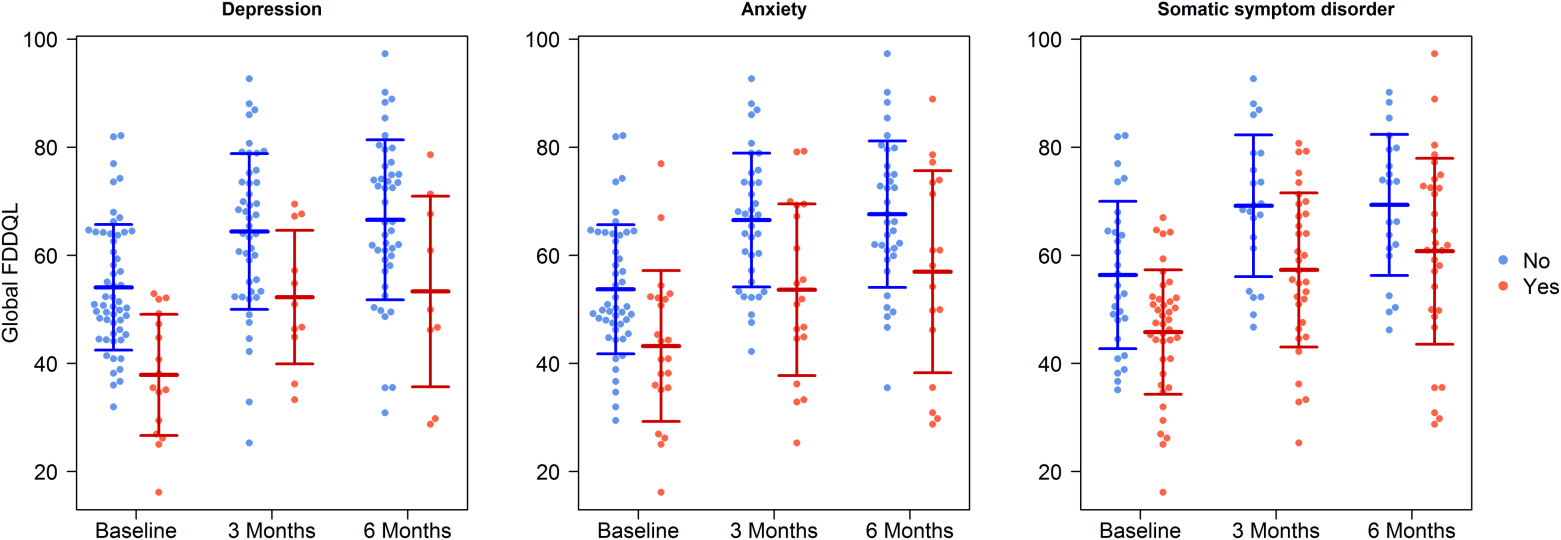
Quality of life (FDDQL) in patients with and without psychosomatic comorbidity.

We observed a significant between-subjects effect on the global FDDQL score related to depression (p = 0.001), anxiety (p = 0.001) and somatization (p = 0.011) (Fig 1). Thus, the quality of life of patients with psychosomatic comorbidity was lower at baseline, but showed a similar increase over the following six months. However, there was no statistically significant interaction between time and depression, anxiety, or somatization (p > 0.100, for each); which means that there was no significant difference regarding the increase in quality of life between the groups with and without these mental disorders (Table 3).

**Table 3:** Interaction effect and between-subjects effect on Global FDDQL.

	Global FDDQL	t1	t2	t3	Interaction effect	Between-subjects effect	Within effect
		Mean (SD)	Mean (SD)	Mean (SD)	F-value (F_crit_)	F-value (F_crit_)	F-value (F_crit_)
**Depression**	no (n = 44)	52.8 (10.0)	63.9 (14.1)	66.6 (14.8)	2.250 (3.323)	13.358 (4.030)	34.447 (3.323)
	yes (n = 9)	32.4 (10.9)	52.8 (13.5)	53.3 (17.6)	**p = 0.211**	**p = 0.001**	**p<0.001**
**Anxiety**	no (n = 35)	53.5 (10.6)	65.9 (12.0)	67.6 (13.5)	0.175 (3.336)	12.404 (4.034)	36.791 (3.336)
	yes (n = 17)	40.6 (12.8)	54.1 (16.7)	57.0 (18.7)	**p = 0.796**	**p = 0.001**	**p<0.001**
**Somatisation**	no (n = 21)	53.5 (11.4)	68.3 (13.0)	69.3 (13.0)	0.346 (3.339)	7.021 (4.034)	41,532 (3.339)
	yes (n = 31)	46.0 (12.7)	57.8 (14.5)	60.8 (17.2)	**p = 0.664**	**p = 0.011**	**p<0.001**

p- and F-value for difference between t1 and t3 (ANOVA with repeated measures)

## Discussion

This study shows that the German version of the self-help guidebook led to a remarkable improvement in quality of life in patients with IBS, which was maintained after six months. This was true for the global score of the FDDQL, and consequently for nearly all subscales with the exception of ´impact of stress´. Beyond that, anxiety, depression and somatization were significantly reduced. Patients with psychiatric comorbidities at baseline showed generally lower FDDQL scores; their observed increase in quality of life was of a similar magnitude as for patients without psychiatric comorbidities.

IBS patients are known to have an impaired quality of life as they face the challenge of dealing with a chronic disease which to date lacks effective treatment. The symptoms affect patients’ everyday lives through an altered self-perception and a need for social and personal re-definition [19]. Self-management is an important treatment approach that has been shown to be effective in helping people with IBS to face these challenges [20]. The best outcomes have been shown for self-management interventions that encourage patients to actively engage, show confidence, self-responsibility and personal initiative [21]. The guidebook evaluated in this study comprises and implements many of the factors known to be crucial for effective self-management. Boger et al. recently defined important goals and outcomes of self-management from patients’ points of view [22]. According to their review, the onset of a chronic disease leads to an urge for physical independence and control over the illness. The self-help guidebook enables these important competencies as it provides comprehensible information on pathogenesis, diet and various treatment strategies. Accordingly, the self-help guidebook facilitates a patient-centred treatment approach that can be implemented and tailored to the individual’s needs. This is of importance, because a problem-oriented, patient-centered approach is known to play a key role in successful self-management support. It is an important prerequisite for an equal physician-patient relationship and thus for shared decision making [23,24].

Psychiatric comorbidities play an important role in IBS patients, because these patients suffer from lower self-esteem and reduced sense of coherence [25]. In line with this, patients suffering from IBS tend to use more problem focused coping and avoidance-oriented behaviour [26]. Moreover, psychiatric comorbidities mediate symptom severity and persistence. They influence the decision to seek treatment and lead to a poorer response to treatment. Accordingly, we initially hypothesized that the self-help guidebook would be less effective in patients with psychiatric comorbidities due to a vicious circle of increased anxiety causing increased perception of pain and visceral sensations, which can result in further symptom exacerbation [27]. However, we observed that while starting at a lower level of quality of life at baseline, the increase in quality of life in participants with depression, anxiety or somatization disorder was of the same magnitude as that of patients without those comorbidities. A possible explanation for our results may be changed cognition and behaviour. Addressing misconceptions, providing structured and reliable information through the guidebook and empowering patients to reinterpret symptoms could possibly lead to a change of affective state by breaking the vicious circle of anxiety, pain and symptom exacerbation. This might ameliorate not only patients’ quality of life, but also improve their mental health [27]. However, applying psychotherapy and medication in IBS patients with psychological comorbidities might address their illness additionally in a more specific way. Further studies have to evaluate the usefulness of a combination of the handbook and psychotherapy or medication in these patients.

### Limitations

We had no control group design because of the pilot character of the study. Thus, we cannot exclude that the observed improvement in quality of life was due to other reasons, like the effect of a regression to the mean [28]. Moreover, the context in which the patients received the guidebook possibly influenced the success of the treatment. Kaptchuk et al. demonstrated the importance of an empathetic doctor-patient relationship with IBS patients in a three-arm randomized controlled trial. They concluded that an intensified doctor-patient relationship and positive expectations are of particular significance in IBS patients [29]. Heightened expectations of study participants leading to an augmented response could therefore be another explanation for the observed results. However, our results are consistent with recent studies indicating that self-administered or minimal contact therapies are effective in IBS [30,31]. The self-help guidebook supports an active engagement by strengthening of self-efficacy and patient empowerment. Since the self-help guidebook implements many elements of self-management concepts known to be effective [30], it seems credible that a causal relationship exists between its use and the increase in quality of life. Beyond that, we implemented no control mechanisms as to the extent the patients used the information provided in the guidebook. It can be assumed that patients who lack successful coping strategies need more information and treatment options. We expected a high motivation and therefore an enhanced willingness to read the handbook. This is in line with the findings of Kennedy et al., that the main reason of the patients for participation in the development of the handbook was their need for information [12]. Documentation of medication was omitted to save workload of the GPs. Therefore, we could not determine the impact of medication use. However, we expect no distortion of our results, because medication for patients with persistent IBS complaints is not very effective.

### Conclusion

The self-help guidebook significantly improved measured quality of life for IBS patients. The use of screening questionnaires like PHQ might be valuable to identify patients with more complex problems. This might be helpful for them to intensify and adapt therapy. Further research has to evaluate, if patients with psychological comorbidity are treated more effectively when they receive psychotherapy or specific medication in addition to the self-management guidebook.

## Supporting information

S1 Fig German version of the self-help guidebook. (PDF)

S1 Table Study data. (XLSX)

S2 Table Description of variables. (DOCX)
