# Supplementary material for: Self-help guidebook improved quality of life for patients with irritable bowel syndrome
Source: PLoS One. 2017 Jul 25;12(7):e0181764. doi: 10.1371/journal.pone.0181764 (PMC5526555; doi:10.1371/journal.pone.0181764)
Supplement: S1 Fig — (PDF) [file pone.0181764.s001.pdf]

Klinikum rechts der Isar
Technische Universität München
Institut für Allgemeinmedizin

Leben mit dem Reizdarmsyndrom – übernehmen Sie die Kontrolle

Ein Selbstmanagement-Handbuch für Patienten

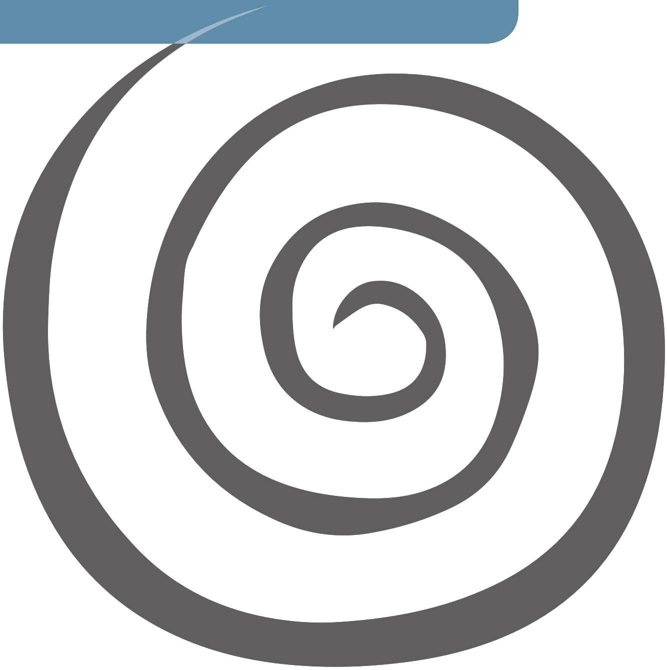

Das Selbstmanagement-Handbuch dient zur freien Verwendung für Patienten.

Das Werk ist urheberrechtlich geschützt.

Jede Verwertung außerhalb der engen Grenzen des Urheberrechtsgesetzes
ist ohne Einwilligung der Autoren nicht zulässig und unter Umständen strafbar.
Das gilt insbesondere für Vervielfältigung, Übersetzungen, Mikroverfilmungen
und EDV-technische Verarbeitung.

Inhalt

Vorwort für das deutsche Selbstmanagement-Handbuch 2

Vorwort von Anne Kennedy 3

Kapitel 1 – Persönliche Erfahrungen mit dem RDS 5

Kapitel 2 – Das RDS verstehen 13

Kapitel 3 – Was Sie tun können, um sich selbst zu helfen 33

Kapitel 4 – Wie man noch mit dem RDS umgehen kann. 51

Kapitel 5 – Ärztliche Behandlung 61

Kapitel 6 – Zusammenfassung und Informationsquellen 67

Hilfreiche Quellen für weitere Informationen 76

Glossar medizinischer Fachbegriffe 77

Literaturverzeichnis 79

Selbsthilfe-Checkliste. 80

Ernährungs-Wochenplan 82

Liebe Patientin, lieber Patient,

das Reizdarmsyndrom ist eine Erkrankung, unter der sehr viele Menschen leiden. Das Leitsymptom sind Bauchschmerzen, die oft mit Blähungen und abwechselnd Durchfall und Verstopfung einhergehen. Manche leiden auch nur unter den Bauchschmerzen mit Blähungsgefühlen, das kann sehr unterschiedlich sein. Obwohl das Reizdarmsyndrom so häufig vorkommt, wurden bisher immer noch keine exakten einheitlichen Ursachen für diese Beschwerden gefunden. Bei vielen Menschen treten wiederholt Bauchschmerzen mit Blähungsgefühlen auf, wenn sie etwas gegessen haben, was sie nicht gut vertragen. Das können bei der sogenannten Laktoseintoleranz Milchprodukte sein. Manche Menschen vertragen auch bestimmte Zusatzstoffe in den Nahrungsmitteln nicht. Andere wiederum reagieren sehr auf Stress oder andere psychische Belastungen – jeder Patient ist da unterschiedlich und hat seine eigene Leidensgeschichte. Oft ist es gerade bei Erkrankungen, die so viele unterschiedliche Ursachen haben können, schwierig, eine effektive Hilfestellung zu leisten. Das Selbsthilfehandbuch ist eines der wenigen Instrumente, bei denen gezeigt werden konnte, dass es Patienten mit Reizdarmsyndrom hilft, besser mit ihren Beschwerden zurecht zu kommen.
Das Selbsthilfehandbuch wurde in England entwickelt. Weil es so hilfreich sein kann, haben wir uns dazu entschlossen, es zu übersetzen und an das deutsche Gesundheitssystem anzupassen. Hierbei haben wir auch wissenschaftliche Neuerungen und die Empfehlungen der S3-Leitlinie „Reizdarmsyndrom“ berücksichtigt. Lesen Sie es in Ruhe durch – es sind für Jeden hilfreiche Tipps dabei. Für die Erlaubnis, das Buch hierfür zu verwenden, bedanken wir uns sehr herzlich bei Frau Professor Anne Kennedy, University of Southampton. Wir hoffen, dass wir damit vielen Menschen helfen können. Wir hoffen, dass Sie diesen Ratgeber verwenden, um

- darüber nachzudenken, wie Sie mit dem RDS umgehen
- darüber nachzudenken, ob Sie etwas verändern wollen
- die Vorschläge in diesem Buch zu nutzen, um Veränderungen leichter umsetzen zu können

Über Rückmeldungen zu dem Handbuch, auch zu Dingen, die wir verbessern könnten, freuen wir uns sehr.

Prof. Dr. med. Antonius Schneider
Institut für Allgemeinmedizin,
Klinikum rechts der Isar,
Technische Universität München

Prof. Dr. Paul Enck
Abteilung Psychosomatik
und Psychotherapie,
Universitätsklinikum Tübingen

Gute Informationen zum Reizdarmsyndrom (RDS) sind wichtig. Sie können Ihnen helfen, zu verstehen, was mit Ihnen passiert, so dass Sie mit den Symptomen und dem Effekt, den das RDS auf Ihr tägliches Leben hat, besser umgehen können. Dieses Buch bietet Ihnen Informationen zu den unterschiedlichen Behandlungsmöglichkeiten, die verfügbar sind, damit Sie die begrenzte Zeit, die Sie mit Ihrem Arzt haben, so gut wie möglich nutzen können. Informationen zu der Krankheit, die auf medizinischer Forschung beruhen, sind sehr wichtig dabei, das medizinische Problem zu verstehen, und herauszufinden, was die besten Behandlungsmöglichkeiten sind, die von medizinischen Einrichtungen angeboten werden. Jedoch haben Menschen, die mit dem RDS leben und damit Erfahrung haben, eine andere Art des Wissens, die genauso wichtig dafür ist, mit der Krankheit im Alltag gut umzugehen und sie zu managen. Forschungsergebnisse dazu, wie die Menschen Krankheit erfahren, legen nahe, dass sie schon viel über die persönlichen und sozialen Auswirkungen der Krankheit wissen, und mit der Zeit werden sie Experten darin, ihre Krankheit zu verstehen und damit umzugehen. Das RDS wirkt sich auf Beziehungen mit anderen Menschen, die Arbeit und Freizeitaktivitäten aus. Menschen mit einem RDS können sehr gut anderen helfen, die sich in einer ähnlichen Situation befinden. Sie wissen, wie es ist, Dinge mit medizinischen Fachleuten zu besprechen, und welche Hilfe man von diesen Kontakten erwarten kann.

Jeder hat seine eigene Theorie dazu, was das RDS verursacht, und diese Theorien verdienen es, als sinnvolle Möglichkeiten anerkannt zu werden, weil man die medizinische Ursache noch nicht sicher weiß. Menschen mit einem RDS haben in der Regel schon eine Bandbreite an Behandlungen und Heilmitteln ausprobiert und haben oft ihre eigene Strategie entwickelt, damit umzugehen. Medizinische Fachkräfte wissen vielleicht nicht gut genug Bescheid darüber, wie es ist, jeden Tag mit dem RDS zu leben. Daher ist es wichtig, mit anderen zu teilen, was Sie hilfreich finden und was nicht. Dieser Ratgeber will das Wissen der medizinischen Fachkräfte und der Menschen mit einem RDS verbinden, um für Sie eine nützliche Quelle zu schaffen. Wir wissen, dass dieser Ratgeber für Menschen mit einem RDS etwas verändern kann, weil er in mehreren großen Studien verwendet wurde und Menschen half, die Art und Weise zu verändern, wie sie über ihr RDS dachten und damit umgingen. Wir haben festgestellt, dass viele das Buch mit ihrer Familie und ihren Freunden teilen, damit andere Menschen besser verstehen, was es bedeutet, mit dem RDS zu leben. Literaturhinweise finden Sie am Ende des Buches.

Anne Kennedy
Principal Research Fellow,
Universität von Southampton, Großbritannien,
Autorin der englischen Fassung

Im Folgenden wird für die bessere Lesbarkeit bei Personenangaben (z.B. Arzt/Ärztin) in der Regel die männliche Form verwendet, damit ist immer auch die weibliche Form gemeint.

Definition Reizdarmsyndrom

Was bedeutet dieses Wort?

Reiz / gereizt: der Darm reagiert sehr sensibel und überschießend auf Verdauungsvorgänge.

Darm: Verdauungsschlauch

Syndrom: eine Gruppe von Symptomen, die einen Krankheitszustand beschreiben.

Wichtiger Hinweis

Wie jede Wissenschaft ist die Medizin ständigen Entwicklungen unterworfen. Forschung und klinische Erfahrung erweitern unsere Kenntnisse, insbesondere was Behandlung und medikamentöse Therapie anbelangt.

Soweit in diesem Handbuch eine Untersuchung, ein Behandlungsverfahren oder eine medikamentöse Anwendung erwähnt wird, wurde von den Autoren größte Sorgfalt verwendet, dass diese Angaben dem aktuellen Kenntnisstand der Forschung bei Fertigstellung des Handbuchs entsprechen.

Es wird jedoch keine Gewähr und Haftung für die Vollständigkeit, Richtigkeit, Genauigkeit und Aktualität sämtlicher Inhalte und Angaben übernommen. Insbesondere kann dieses Handbuch nicht für die eigenständige Selbstdiagnose dienen.

Sie sollten sich wegen Ihrer Verdauungsbeschwerden auf jeden Fall bei einem Hausarzt, Internisten oder Magen-Darm-Spezialisten vorstellen. Das Handbuch ersetzt nicht den Arztbesuch.
Dieses Handbuch soll Ihnen als Ratgeber dienen, wenn bei Ihnen tatsächlich ein Reizdarmsyndrom vorliegt.

Kapitel 1

Persönliche Erfahrungen mit dem RDS

„A good set of bowels is worth more to a man than any quantity of brains.“
Josh Billings

(Sinngemäß: Für das Wohlbefinden ist der Darm wichtiger als das Gehirn.)

Dieser Ratgeber wurde mit der Hilfe von Menschen geschrieben, die ein Reizdarmsyndrom (RDS) haben. Menschen mit einem RDS trafen sich in mehren Gruppen, um über ihre Probleme zu sprechen und zu entscheiden, welche Art von Informationen sie für besonders hilfreich halten. Die Zitate in diesem Handbuch sind von Menschen mit einem RDS, die zu diesen Treffen kamen. Alle waren der Meinung, dass es ihnen sehr half, über ihr RDS zu sprechen. Was sie vor allem wissen wollten war, wie andere ihr Leben bewältigen. In diesem ersten Kapitel werden Sie Berichte von Menschen finden, die mit einem RDS leben. Sie werden wahrscheinlich feststellen, dass Sie viele Gefühle und Erfahrungen gemeinsam haben, und es wird Ihnen hoffentlich zeigen, dass Sie nicht alleine sind.

Viele Menschen haben ein RDS. Es ist schwierig, genaue Zahlen zu nennen, da viele Menschen nicht ihren Arzt um Rat fragen, aber es wird vermutet, dass sogar einer von zwei Menschen zu einer gewissen Zeit im Leben Symptome eines RDS hat. Die Symptome kommen und gehen, und die meisten Menschen kämpfen, um mit ihnen umgehen zu können. In gewissem Sinne ist es also auch ganz normal, dass der Darm einmal nicht so gut funktioniert. Das, worauf es ankommt, ist das Ausmaß der Störung. Dies kann vom unangenehmen Blähungsgefühl bis hin zu fast nicht kontrollierbarem Stuhlgang reichen.

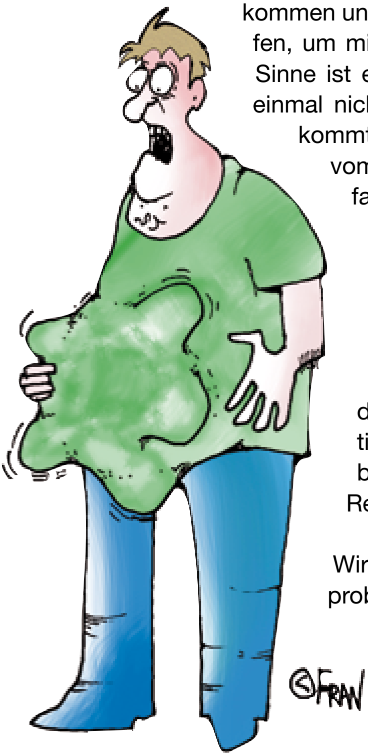

Menschen mit einem RDS haben einen Darm, der nicht richtig funktioniert. Das RDS besteht aus einer Reihe von Symptomen, und wir wissen, dass manche Dinge es verschlimmern können. Es ist eine Erkrankung, die die Menschen auf viele Arten beeinträchtigt. Dieser Ratgeber möchte Ihnen Tipps geben, die Ihnen hoffentlich helfen werden, Ihre Reizdarmsymptome zu kontrollieren.

Wir alle finden es peinlich, über Verdauungsprobleme zu sprechen. Das RDS kann eine einsame und beunruhigende Erfahrung sein.

„Es ist keine ernste Krankheit, aber es ist eine komplizierte Krankheit. Ein riesiges Organ, das verrückt spielt, und es scheint keine Heilung zu geben."

„Das Entscheidende ist, herauszufinden, dass es eine richtige Krankheit ist. Wissen Sie, es ist nicht etwas, das ich mir ausgedacht habe. Ich glaube, es hilft, zu wissen, dass es auch andere Menschen gibt."

Da der Darm aus so vielen verschiedenen Teilen besteht, haben Menschen mit einem RDS viele Symptome. Dazu können Verstopfung, Durchfall und Schmerzen gehören. Es kann sein, dass Sie immer die gleichen Symptome haben, oder Sie können feststellen, dass sich Ihre Symptome verändern. In den allermeisten Fällen verursacht das RDS keine ernsthaften Probleme.

„Es kann sein, dass so… ein, zwei Monate alles gut läuft und dann plötzlich… Es fängt mit starker Verstopfung an, dann die Darmkoliken und das auf die Toilette gehen. Am nächsten Tag fühle ich mich, als ob jemand auf mir herum gesprungen wäre."

„Ich bekomme keine Verstopfung. Ich muss jeden Tag, aber ich habe danach nicht das Gefühl, dass ich auf der Toilette war. Ich habe immer noch das Gefühl, dass mein Bauch richtig aufgebläht ist."

„Es flammt wahrscheinlich so zwei oder drei mal im Jahr auf. Ich habe dann oft starken Durchfall, fühle mich aufgebläht und mir ist übel."

„Genau wie Regelschmerzen, ich wälze mich auf dem Boden und ich fühle mich aufgebläht und die Blähungen sind fürchterlich."

„Es hat Nebenwirkungen, nicht nur den Schmerz – man ist aufgebläht, man fühlt sich lustlos. Ich kriege Kopfschmerzen."

„Man fühlt sich einfach wie ein nasser Waschlappen. Es macht einen einfach kaputt."

Die Menschen haben sehr ähnliche Gefühle und Sorgen, wenn sie Verdauungsprobleme haben.

„Ich habe mir zunächst Sorgen gemacht, dass ich Darmkrebs hätte, die Schmerzen waren so schrecklich, ich war mir sicher, dass da irgendwas schlimmes war, und ich hatte diese Koloskopie und sie haben nichts gefunden."

„Mir fällt es schwer, das zu glauben, es kann plötzlich einfach so kommen und man wird es nicht los. Es muss doch etwas geben, das man tun kann, um damit besser umgehen zu können, oder?"

Eines der Probleme des RDS ist der Kontrollverlust, den die Menschen spüren.

„Es ist so unvorhersehbar. Im einen Moment geht es einem gut und im nächsten geht es los."

„Ich suche mir die Toiletten heraus, bevor ich irgendwo hingehe. Ich muss einfach wissen, wo die Toiletten sind."

„Ich habe das Gefühl, dass ich keine längeren Fahrten planen kann, wenn ich es habe. Ich würde nicht versuchen, weiter als bis zum nächsten Supermarkt zu fahren."

„Wenn ich weggehe, nehme ich schachtelweise Einlagen und Feuchttücher mit, weil ich manchmal einfach nicht vorgewarnt werde."

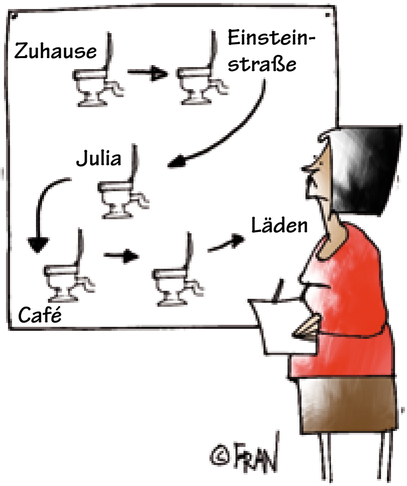

Niemand weiß, was das RDS verursacht – und nicht zu wissen, was eine Krankheit auslöst, kann frustrierend und beängstigend sein. Für die meisten, aber nicht für alle, ist es eng mit Stress verbunden. Ob Stress die Ursache ist oder

nicht, das RDS wird wahrscheinlich schlimmer, wenn man sich in einer stressigen Situation befindet.

> ***„Ich glaube, durch Stress kann es bei mir losgehen. Ich habe Probleme in der Arbeit, ich kann mich da richtig drin festbeißen – das kann es bei mir auslösen."***

> ***„Ich weiß, dass ich Panik kriege wenn mein Freund einen Anfall hat, ich kriege Panik und rege mich richtig auf und dann, am nächsten Tag, den ganzen Tag auf der Toilette."***

> ***„Ich habe mich früher leichter in etwas festgebissen, ich glaube ich habe versucht, mich zu beweisen. In letzter Zeit bin ich mehr mit mir im Reinen – also ob das geholfen hat, dass es bei mir ein bisschen besser läuft..."***

Bei manchen Menschen scheint Stress nicht die Ursache des RDS zu sein.

> ***„Ich kriege es eher, wenn ich mich entspanne, also diese Theorie mit dem Stress, ich glaube nicht, dass die wirklich stimmt."***

Manche Menschen verbinden den Beginn des RDS mit anderen Ursachen.

> ***„Ich hatte Probleme mit der Pille und ich habe die Pille gewechselt – danach hat es angefangen. Es könnte hormonell sein, das könnte es ausgelöst haben."***

Essen und Ernährungsgewohnheiten sind eng mit dem RDS verbunden. Fast jeder mit einem RDS kennt Nahrungsmittel, die bei ihm Symptome auslösen. Manchmal kann man definitiv eine Verbindung zwischen einer schlechten Erfahrung mit Lebensmitteln und dem Beginn des RDS herstellen.

> ***„Als ich dieses Truthahnfleisch gegessen habe, dachte ich, „Oh Gott", es hatte einen sehr kräftigen Geschmack. Am nächsten Tag begann ich, mich krank zu fühlen, ich war eine ganze Woche krank. Es wurde besser, aber es ist nie mehr ganz weggegangen, es kam wieder und wieder."***

„Ich mache einfach eine Pause mit Dingen wie Salat und Obst, wenn es schlimm ist. Man lernt einfach aus Erfahrung, man denkt, „Das esse ich wohl nicht mehr“, wenn man sich aufgebläht fühlt.“

Die Menschen können viele Wege finden, mit den Problemen, die das RDS mit sich bringt, umzugehen. Manche können mit ihrem RDS alleine umgehen und es selbst managen.

„Die ersten Symptome hatte ich vor fast 25 Jahren, und ich habe alles durch, was der Arzt mir anbieten konnte. Ich habe schließlich selbst eine Lösung gefunden, indem ich meine Essgewohnheiten verändert habe, das war die Lösung für mich.“

Bei den meisten wird das RDS mit der Zeit besser. Das kann daher kommen, dass man mehr darüber lernt, wie der Körper auf Essen oder stressige Ereignisse reagiert. Man findet Wege, damit umzugehen.

„Jetzt, wo ich älter bin, ist es besser kontrollierbar.“

„So langsam ist es nicht mehr so schlimm wie am Anfang.“

„Ich glaube, es ist ein langer Weg zur Selbsthilfe. Man muss realistisch sein und denken: „Was verursacht das?“, und einen Weg finden, das zu umgehen.“

Was manchen auch zu helfen scheint, ist, über das Problem zu sprechen. Das RDS ist eine sehr häufige Krankheit. Es ist sehr wahrscheinlich, dass andere aus Ihrem Kollegen- oder Freundeskreis auch unter einem RDS leiden.

„Als ich meine Stelle dort antrat und die ganzen Leute gefunden habe, die es auch haben, war ich sehr überrascht, aber es hilft wirklich, mit anderen Leuten zu reden.“

Das RDS verursacht Probleme und beeinträchtigt das Leben. Studien haben gezeigt, dass es ein häufiger Grund für Fernbleiben vom Arbeitsplatz ist.

> *„Ich habe fast diese Stelle verloren. Jedes mal, wenn ich zuhause blieb und eine Bescheinigung wegen des RDS brachte, musste ich ins Sekretariat, weil ich mit Lebensmitteln arbeitete. Es war sehr peinlich, sie saßen da und bombardierten mich mit Fragen. Ich sagte nur, dass ich ständig auf die Toilette muss, ich bin nicht schmutzig, jeder muss auf die Toilette gehen, ich eben öfter.“*

Es verursacht oft Probleme im Sozialleben.

> *„Ich weiß noch, dass ich mal auf einer Hochzeit war. Ich trug einen sehr enganliegenden Rock mit einer Bluse, die ich reingesteckt hatte. Es war die Hölle. Der Rock sah schrecklich aus, weil mein Bauch so heraus-stand. Ich hatte die ganze Nacht schreckliche Schmerzen, ich konnte nichts vom Buffet essen.“*

> *„Letzten Monat bin ich in die Stadt gefahren. Ich bin in einem großen Kaufhaus auf die Toilette gegangen, es gab eine lange Schlange, ich habe mich eingesaut. Ich musste mir eine Jogginghose kaufen gehen. Ich kam bei meiner Tochter an und war so aus der Fassung, dass ich es ihr nicht erzählen konnte. Ich habe mich sauber gemacht und mich gefragt, ob die Leute mich riechen können. Das zieht einen ganz schön runter.“*

> *„Wegen meines Bauchs habe ich seit drei Jahren keinen Urlaub mehr gemacht.“*

Die Mitmenschen können sehr verständnislos sein. Dann fühlt man sich oft schrecklich.

> *„Eine meiner Nachbarinnen war diesen Frühsommer schwanger und sagte: „Du bist ja ungefähr so rund wie ich, oder?“ Ich hätte sterben können vor Peinlichkeit, weil unsere Bäuche ziemlich ähnlich aus-sahen.“*

Die Erfahrungen mit Ärzten sind ganz unterschiedlich. Menschen, die ein RDS schon seit einiger Zeit haben, haben oft das Gefühl, dass sie alles versucht haben, was ihre Ärzte vorgeschlagen haben, und ver-suchen, selbst damit fertig zu werden.

„Ich war schon ewig nicht mehr bei meinem Hausarzt. Ich habe das Gefühl, dass er mir nichts erzählen kann, was ich nicht eh schon weiß und ausprobiert habe.“

„Der Arzt hat mir gesagt: „Es gibt keine Heilung für das RDS. Sie müssen lernen, damit umzugehen.“ Und ich glaube, das stimmt.“

Manche verlassen sich auf die emotionale Unterstützung ihres Arztes. Manche Ärzte schaffen das, andere nicht. Es kann sehr schwierig sein, wenn Sie das Gefühl haben, ihr Problem nicht mit dem Arzt besprechen zu können. Es kann sein, dass es Ihnen besser geht, wenn Sie mit jemand anderem sprechen, zum Beispiel einem Therapeuten, oder jemandem, der auch ein RDS hat.

„Wenn man jemanden hat, der einfühlsam ist, und der sich damit auskennt, und der lächelt, anstatt zu denken, „Der nächste bitte“, wissen Sie, das ist echt gut.“

„Manchmal rede ich darüber und breche in Tränen aus und der Arzt schaut mich nur an, ich wette er denkt, „Oh, die schon wieder“, und ich will am liebsten sagen: „Bitte, kann mir nicht irgendwer helfen, gebt mir doch einfach ein bisschen ein schönes Leben.““

„Wenn sie sagen: „Das ist Kopfsache“, das ist etwas, das mir richtig zusetzt, ich denke dann: „Nein, ist es nicht, es ist eigentlich Bauchsache.““

„Ein Arzt ist nicht das gleiche wie ein Therapeut, man muss sich hinsetzen und das mit jemandem besprechen, der sich wirklich anhört, was einen beschäftigt, und nicht nur denkt, „Sie kriegen Tabletten“, oder so.“

„Man braucht einen Therapeuten mit einem RDS. Das ist die Lösung.“

Denken Sie daran …

… Viele Menschen haben ein RDS.
… Man kann viele unterschiedliche Symptome haben.
… Es kann schwer sein, mit einem RDS zurechtzukommen, aber es ist nicht lebensgefährlich.

Kapitel 2

Das RDS verstehen

*Dieses Kapitel erklärt Ihnen,
was man über das RDS und
über die normale Verdauung weiß
und was aktuelle Studien zum RDS zeigen.*

Was bedeutet Reizdarmsyndrom?

Das RDS ist schon seit langer Zeit bekannt. Es hatte schon viele Bezeichnungen, wie z.B.: Colon irritabile, Reizkolon und „nervöser Darm“. Zwischen 2,5% und 25% der Bevölkerung haben ein RDS, die genaue Anzahl ist nicht bekannt, weil es viele Definitionen des RDS gibt.
In Deutschland gehen jedes Jahr 5 bis 10% der Bevölkerung wegen eines RDS zum Hausarzt. Das RDS ist eine Kombination aus Bauchschmerzen und veränderten Stuhlgewohnheiten. Die Ursache ist, dass der Darm nicht normal kontrahiert (sich zusammenzieht) und sehr sensibel auf die Kontraktionen reagiert.
Frauen haben dreimal so häufig ein RDS wie Männer. Normalerweise beginnt es in der späten Jugendzeit, aber es kann in jedem Alter anfangen.
Wenn man Menschen mit einem RDS untersucht, sieht ihr Darm gesund aus und die Blutuntersuchungen zeigen nichts Ungewöhnliches. Aber trotzdem funktioniert ihr Darm nicht richtig. Die Forschung hat gezeigt, dass das RDS keinen Krebs verursacht.

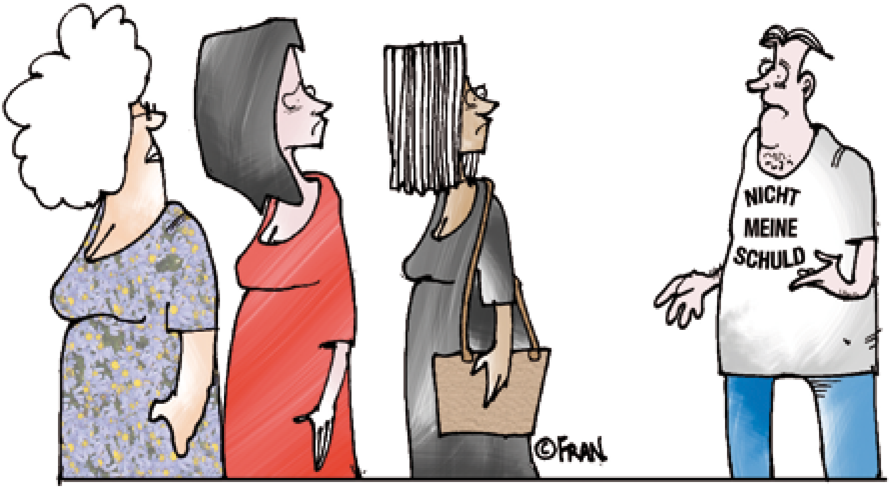

Manche Patienten sind erleichtert, dass die Untersuchungen kein ernstes Problem in ihrem Körper zeigen. Andere finden die Nachricht „alles ist in Ordnung“ sehr frustrierend und wünschen sich eine eindeutige körperliche Ursache.

Was meinen die Ärzte mit dem Wort „Syndrom“?

Ein Syndrom ist eine Gruppe von Symptomen, die einen Krankheitszustand ausmachen. Um das RDS zu diagnostizieren, muss ein Arzt wissen, ob Sie zwei oder drei der folgenden Symptome haben:

- Bauchschmerzen
- Durchfall
- Verstopfung
- Blähungen
- Einen Blähbauch
- Das Gefühl, dass der Darm nicht komplett entleert ist
- Schleimbeimengungen

Oft hat man nach dem Essen Schmerzen, die durch Darmentleerung häufig besser werden.

Dies sind KEINE Symptome eines RDS

- Blutbeimengungen oder
- Gewichtsverlust

Wenn Sie eines dieser Symptome feststellen, müssen Sie so schnell wie möglich zum Arzt gehen, weil das Symptome einer entzündlichen Darmerkrankung oder von Darmkrebs sein könnten. Wenn Sie über 40 Jahre alt sind und das erste Mal Reizdarmsymptome haben, sollten Sie Ihren Hausarzt aufsuchen, der möglicherweise weitere Untersuchungen veranlassen wird.

! Wenn Sie Blut ausscheiden oder ohne Diät mehr als 6 kg abgenommen haben, wird Ihr Arzt Sie wahrscheinlich zu weiteren Untersuchungen schicken, um ein ernsteres Darmproblem auszuschließen.

Medizinische Forschung

Es wird geforscht, um die folgenden Dinge über eine Erkrankung herauszufinden:
- Die Anzahl der Betroffenen und ob diese Zahl steigt
- Wer die Krankheit bekommt
- Welche Symptome die Krankheit verursacht
- Was im Körper von Erkrankten nicht normal ist
- Was die Symptome verbessert oder verschlechtert
- Wie sicher die Behandlungsmethoden sind

Für das RDS können wir diese Fragen beantworten, indem wir mit betroffenen Patienten sprechen, den Darm auf Abnormalitäten untersuchen, und indem wir neue Behandlungsmethoden in klinischen Studien ausprobieren.
Bis jetzt zeigt die Forschung, dass es keine eindeutigen körperlichen Unterschiede zwischen Patienten mit einem RDS und Menschen ohne die Krankheit gibt. Es wurde viel Forschung zu den unterschiedlichen Behandlungsmethoden betrieben.
In klinischen Studien wurden verschiedenste Medikamente, Psychotherapie, Hypnotherapie, und komplementäre Therapien (u.a. Naturheilverfahren) getestet. Viele Studien haben unklare Ergebnisse und beziehen sich auf Patienten mit sehr spezifischen Symptomen. Es ist schwierig, grundsätzliche Schlussfolgerungen zu ziehen, aber es gibt keine Beweise dafür, dass eine einzige Behandlungsmethode nützlich für alle Menschen mit einem RDS ist.
Gute wissenschaftliche Studien sind teuer und es ist oft schwer, Geld für die Forschung zu bestimmten Krankheiten zu bekommen. Durch die medizinische Forschung gewinnen wir jedoch einige Theorien und Konzepte zu einzelnen Krankheitsbildern. In den nächsten Abschnitten werden Sie mehr zum RDS und dessen Ursachen erfahren, basierend auf aktuellen Forschungsergebnissen.

Erste Theorie

Etwas in Ihrer Nahrung könnte dazu führen, dass Ihr Darm krankhaft reagiert. Ist es eine Infektion? Ist es eine Lebensmittelunverträglichkeit, weil Ihrem Körper ein bestimmter chemischer Stoff fehlt? Sind es unsere modernen Ernährungsgewohnheiten? Ist es eine allergische Reaktion?

>> Die Forschungslage: Einige Forschungsergebnisse haben gezeigt, dass es eine Verbindung zwischen dem RDS und Menschen gibt, die im Laufe ihres Lebens viele Antibiotika einnehmen. Studien haben gezeigt, dass mindestens ein Viertel der Menschen, die ihren Arzt mit einem RDS aufsuchen, eine Magen-Darm-Infektion hatte, bevor die Symptome begannen. Es gibt starke wissenschaftliche Hinweise darauf, dass manche Menschen, bei denen ein RDS diagnostiziert wird, eine Laktoseintoleranz haben könnten, also bekommen sie nur Reizdarmsymptome, wenn sie Milchprodukte essen oder trinken. Es gibt kaum Hinweise, die zeigen, dass Lebensmittelallergien eine Ursache von Reizdarmsymptomen sind.

Zweite Theorie

Es gibt eine Theorie, die besagt, dass die aktuelle Zunahme von Lebensmittelunverträglichkeiten damit zusammenhängt, dass wir weniger Bakterien ausgesetzt sind. In der westlichen Welt sind die Häuser heutzutage viel sauberer als früher. Wir benutzen viel mehr Desinfektionsmittel, um unsere Häuser zu putzen, und damit könnte die steigende Zahl an Berichten über Menschen zusammenhängen, die „allergisch“ gegen die moderne Welt sind. Es könnte sein, dass der menschliche Körper zu Beginn des Lebens vielen Bakterienarten ausgesetzt sein muss, um gesund zu bleiben. Diese Theorie konnte man bisher nicht wissenschaftlich beweisen, aber sie wurde auch nicht wissenschaftlich widerlegt.

Dritte Theorie

Es könnte ein Ungleichgewicht zwischen den Bakterienarten im Darm bestehen. Es ist normal, viele Bakterienarten im Darm zu haben, die helfen, das Essen richtig zu verdauen. Manchmal wird das Gleichgewicht durch einen Auslöser, wie z.B. eine Infektion oder Antibiotikaeinnahme, verändert, und es gibt nicht mehr genug hilfreiche Bakterien. Das wissen wir, weil manche Menschen mehr Reizdarmsymptome bekommen, wenn sie Antibiotika (die Bakterien abtöten) einnehmen.

Es gibt auch Hinweise darauf, dass es gegen manche Symptome eines RDS, wie zum Beispiel einen Blähbauch oder Blähungen, helfen kann, hilfreiche Bakterien einzunehmen, zum Beispiel mit speziellen Joghurts oder verkapselten Tabletten. Mehr Informationen dazu gibt es im Kapitel über Ernährung.

Vierte Theorie

Möglich ist, dass sich die Dickdarmmuskeln von Menschen mit einem RDS nicht richtig zusammenziehen. Es könnte sein, dass Menschen mit Verstopfung einen Dickdarm haben, in dem es nicht zu genügend Muskelkontraktionen kommt. Menschen, die Durchfall haben, könnten einen Dickdarm haben, in dem sich die Muskeln zu oft zusammenziehen.

>> Die Forschungslage: Forschung zur Darmmuskulatur beinhaltet das Messen des Drucks im Darm. Das kann man über mehrere Stunden hinweg tun, während die Menschen ihren normalen Tätigkeiten nachgehen und dabei die speziellen Geräte mit sich herumtragen, die man braucht, um die Druckänderungen aufzuzeichnen. Einige Forschungsergebnisse zeigen, dass es zwischen Menschen, die ein RDS mit Verstopfung haben und Menschen, die ein RDS mit Durchfall haben, und gesunden Testpersonen Unterschiede bezüglich der Anzahl und der Stärke der Muskelkontraktionen gibt. Wir wissen noch nicht, was zu diesen Unterschieden führt.

Fünfte Theorie

Es könnte sein, dass etwas mit der Art und Weise nicht stimmt, wie der Darm die Nährstoffe oder sonstige Reize wahrnimmt oder versteht was passiert, während das Essen durch ihn hindurch wandert. Daraus folgt, dass der Darm nicht normal reagiert, was zu den Reizdarmsymptomen führen kann. Der Darm hat sein eigenes Nervensystem, das mit dem Gehirn verknüpft ist.

>> Die Forschungslage: Die Forschung hat gezeigt, dass manche Menschen mit einem RDS einen überempfindlichen Darm haben. Studien, in denen spezielle Ballons in den Darm eingebracht werden, zeigen, dass Menschen mit einem RDS Druckunterschiede viel leichter spüren als Menschen, die kein RDS haben. In der aktuellen Forschung wurden die Nervenverbindungen vom Darm zum Gehirn betrachtet und die Art und Weise, wie das Gehirn darauf reagiert, was im Darm passiert. Wir verstehen noch nicht, ob die Probleme auf der Ebene

des Darms liegen, indem das RDS zu sensibleren Nervenendigungen führt, oder auf Ebene des Gehirns, das Schmerz im Darm registriert, wenn keiner da sein sollte. Ergebnisse zeigen auch, dass unsere Gefühle die Empfindungen aus dem Darm beeinflussen können.

Sechste Theorie

Emotionale Probleme können Darmprobleme verursachen. Das passiert jedem. Wir alle bekommen vor einem mit Stress verbundenen Ereignis wie einer Prüfung oder einem Vorstellungsgespräch ein Kribbeln im Bauch und viele Menschen bekommen Durchfall oder übergeben sich. Vielleicht ist bei Menschen mit einem RDS die emotionale Verbindung zum Darm stärker.

>> Die Forschungslage: Es wurde gezeigt, dass das RDS ein Weg des Körpers ist, physisch auf Stress zu reagieren. Andere körperliche Anzeichen für Stress sind Spannungskopfschmerzen, eine schlechte Widerstandsfähigkeit gegenüber Erkältungen und Virenerkrankungen, und hoher Blutdruck.

Forscher haben eine Verbindung zwischen mit Stress verbundenen Ereignissen und Reizdarmsymptomen gefunden.

Siebte Theorie

Das RDS könnte mit den Hormonen zusammenhängen. Es ist bekannt, dass ein RDS häufiger bei Frauen als bei Männern diagnostiziert wird.

>> Die Forschungslage: Es gibt Forschungsergebnisse, die zeigen, dass Durchfall- und Verstopfungssymptome mit dem Menstruationszyklus zusammenhängen. Man hat herausgefunden, dass Frauen mit einem RDS während der Regelblutung mehr Probleme mit diesen Symptomen haben als Frauen, die kein RDS haben. Bestimmte Hormone, die am ersten Tag der Periode hohe Spiegel aufweisen, sind bekannt dafür, die Muskelkontraktionen des Darms zu erhöhen.

! Es kann sein, dass die Ursache des RDS auf einige, keine oder eine Kombination dieser sieben Theorien zurückzuführen ist.

Die Verdauungsorgane:

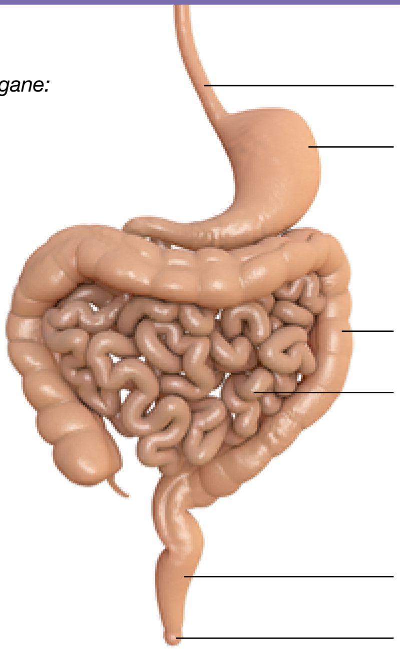

© crevis/fotolia.com

Wie funktioniert das Verdauungssystem?

Wenn Sie wissen, wie das Verdauungssystem funktioniert, kann Ihnen das helfen, das RDS besser zu verstehen.

Der Gastrointestinal- oder Verdauungstrakt ist ein hohler Schlauch, der vom Mund bis zum After reicht. Er verdaut Nahrung, und Nährstoffe werden durch die Darmwand ins Blut aufgenommen. Stoffe, wie zum Beispiel Speichel, Schleim und Enzyme, werden in den Darm freigesetzt, um bei der Verdauung und dem Transport des Essens zu helfen. Unter Verdauung versteht man den Vorgang, bei dem Essen und Getränke in ihre kleinsten Bestandteile zerlegt werden, die man als Nährstoffe bezeichnet. Der Körper verwendet Nährstoffe, um Zellen zu bilden und zu ernähren, und um Energie zu gewinnen. Verdauung beginnt im Mund wenn wir kauen und schlucken, und endet im Dünndarm. Der unverdaute Rest landet im Dickdarm.

Aus dem Mund wandert das Essen weiter durch die Speiseröhre in den Magen, wo es zerkleinert und verdaut wird. Dann wandert das Essen in den Dünndarm. Der Dünndarm ist ungefähr 6,5 Meter lang und wird dünn genannt, weil der Schlauch hier enger ist. Das meiste Essen wird im Dünndarm verdaut und aufgenommen, der aus drei Abschnitten besteht: dem Duodenum (Zwölffingerdarm), dem Jejunum (Leerdarm) und dem Ileum (Krumm- oder Hüftdarm).

Das Ileum ist mit dem Dickdarm verbunden. Der Dickdarm ist ungefähr 1,5 Meter lang und wird dick genannt, weil der Schlauch hier weiter ist. Der Dickdarm verbindet den Dünndarm mit Rektum (Mastdarm) und After. Im Dickdarm wird Flüssigkeit ins Blut aufgenommen und die Nahrungsmittelreste werden in festen Stuhl (Fäzes) umgewandelt. Ungefähr zwei Liter Flüssigkeit kommen jeden Tag vom Dünndarm in den Dickdarm. Diese Flüssigkeit kann dort stunden- oder tagelang bleiben. Während dieser Zeit werden der Großteil der Flüssigkeit und Salze in den Körper aufgenommen. Der Stuhl wandert durch den Dickdarm ins Rektum, wo er verbleibt, bis man auf die Toilette geht.

Die Hohlorgane des Verdauungssystems enthalten Muskeln, die die Darmwände bewegen, indem sie sich zusammenziehen und wieder entspannen.

Die Wandbewegung befördert Nahrung und Flüssigkeiten durch das Verdauungssystem und kann auch den Inhalt des jeweiligen Organs vermischen. Diese Bewegungen der Speiseröhre, des Magens und des Darms nennt man Peristaltik. Man kann sie sich wie eine Welle vorstellen, die sich durch den Muskel bewegt. Der Muskel des Organs zieht sich zusammen und das setzt sich langsam über die Länge des Organs fort. Diese Kontraktionswellen schieben Nahrung und Flüssigkeiten durch den Darm vor sich her. Dickdarmmuskeln bewegen den Inhalt langsam vorwärts in Richtung Rektum. Ein paarmal am Tag bewegen sich starke Muskelkontraktionen entlang des Dickdarms, die Stuhl vor sich herschieben. Wegen mancher dieser Kontraktionen muss man auf die Toilette gehen.

Der Zusammenhang zwischen Gehirn und Darm

In den Verdauungsorganen enden Nervenfasern aus dem Gehirn und dem Rückenmark. Diese Nerven setzten chemische Stoffe frei, die entweder die Muskeln der Verdauungsorgane veranlassen, mit mehr Kraft zu drücken und das Schieben der Nahrung und der Verdauungssäfte durch den Verdauungstrakt zu verstärken, oder sie führen zur Entspan-

nung der Muskeln. Es gibt auch ein dichtes Netz von Nerven in den Wänden von Speiseröhre, Magen, Dünndarm und Dickdarm. Diese Nerven lösen das Zusammenziehen der Muskeln aus, wenn Nahrung die Organwände dehnt. Sie setzen viele verschiedene Substanzen frei, die die Fortbewegung der Nahrung beschleunigen oder verlangsamen, und kontrollieren, wann die Verdauungsorgane Enzyme produzieren.

Normaler Stuhlgang

Wie häufig ist normal?

Die meisten Menschen haben nicht nur einmal am Tag Stuhlgang. In der westlichen Welt, zum Beispiel in Deutschland, kann „normal" alles von dreimal am Tag bis einmal pro Woche bedeuten. Tatsächlich kann es sich von Tag zu Tag ändern, wie oft man Stuhlgang hat. Die häufigste Zeit für Stuhlgang ist kurz nach dem Aufwachen oder nach einer schweren Mahlzeit.

Wie sollte er aussehen?

Ärzte stufen die Stuhlform von 1 bis 7 wie folgt ein:

1. Wässrig
2. Breiig
3. Weich
4. Wurst- oder bananenförmig
5. Wurst- oder bananenförmig aber mit Rissen in der Oberfläche
6. Klumpig und fest
7. Getrennte, harte Klumpen wie Erdnüsse oder Schafskot

Stufen 2 bis 6 gelten als normal.

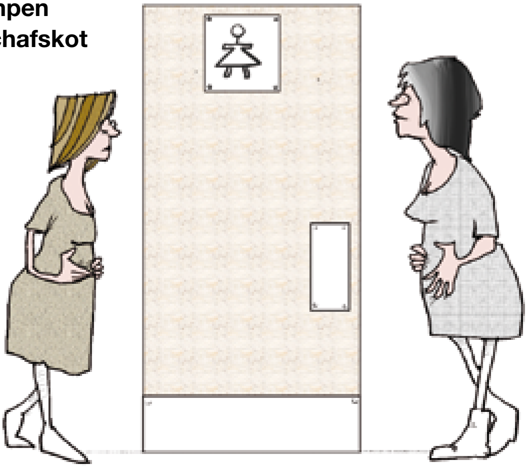

Gasproduktion

Im normalen Verdauungssystem variiert die Gasmenge, die durch den After entweicht, zwischen 500 ml bis zu 1500 ml pro Tag. Bei gesunden Menschen entweichen ungefähr 14 Mal am Tag ca. 25 ml bis 100 ml Gas. Studien haben gezeigt, dass bei Menschen mit einem RDS die gleiche Gasmenge entweicht wie bei Gesunden. Es kann sein, dass die Darmmuskulatur beim RDS sehr sensibel auf das Vorhandensein von Gas reagiert, was zu unangenehmen Symptomen wie Aufstoßen oder Rülpsen, einem Blähbauch, lauten Darmgeräuschen und Blähungen führen kann.

Es befinden sich verschiedene Gase in unterschiedlichen Anteilen des Darms. Der Magen und der obere Darmabschnitt enthalten Sauerstoff, Stickstoff und Kohlendioxid. Sauerstoff und Stickstoff kommen aus der Luft, die man schluckt. Kohlendioxid wird im Magen produziert. Der Dickdarm enthält Wasserstoff, Methan und Kohlendioxid. Dieses Gas wird von Bakterien im Dickdarm produziert, die die Nahrungsmittelreste vergären.

Ernährung und das Verdauungssystem

Grundsätzliche Ratschläge zur Ernährung

Der menschliche Körper braucht eine ausgewogene Ernährung, um gut zu funktionieren. Falls Sie die Form des RDS haben, bei der Ihre Symptome mit dem zusammenhängen, was Sie essen, lassen Sie nicht so viele Nahrungsmittel weg, dass Ihre Ernährung einseitig wird. Es gibt keine allgemeingültigen Regeln zur Ernährung, aber grundsätzlich ist es gut,

- **Mahlzeiten zu regelmäßigen Zeiten einzunehmen**
- **eine große Vielfalt an Nahrungsmitteln zu essen**
- **das, was man isst, an die körperliche Aktivität anzupassen und**
- **ein gesundes Körpergewicht anzustreben.**

Gesundheit durch Balance

Die offiziellen Richtlinien, die die Ernährung in der Gesamtbevölkerung verbessern sollen, empfehlen:

- mehr und eine größere Vielfalt an Obst und Gemüse zu essen
- weniger Fett – besonders gesättigte Fettsäuren – zu essen
- mehr Kohlenhydrate zu essen
- mehr Ballaststoffe zu essen
- weniger Salz und Zucker zu essen und
- Alkohol höchstens in moderaten Mengen zu trinken

Es wurde viel daran geforscht, welche Effekte die Ernährung auf Menschen mit einem RDS hat. Bei einem großen Teil der Forschung wurden die Patienten auf eine Diät gesetzt, bei der sie bestimmte Nahrungsmittel nicht essen durften. Das wird als Ausschlussdiät bezeichnet. Das „erlaubte“ Essen ist sehr schonend und ist dafür bekannt, das Verdauungssystem nicht zu stören. Falls durch diese Diät die Reizdarmsymptome aufhören, werden eins nach dem anderen alle Nahrungsmittel wieder in die Diät mit eingeschlossen. Durch gewissenhafte Aufzeichnungen über die Symptome der Menschen auf dieser Diät haben Wissenschaftler herausgefunden, dass bei manchen Menschen mit einem RDS bestimmte Nahrungsmittel Symptome verursachen.

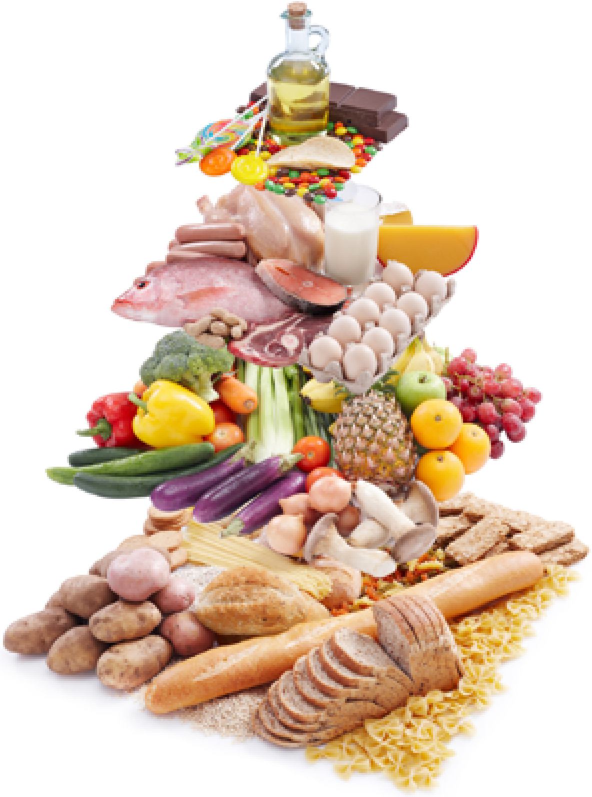

© Okea/Fotolia.com

Die häufigsten Nahrungsmittel, die Symptome verursachen, sind nach Häufigkeit aufgeführt:

- **Milchprodukte (Milch, Käse und Butter)**
- **Nüsse**
- **Tee und Kaffee**
- **Schokolade**
- **Zitrusfrüchte**
- **Eier**
- **Kartoffeln**
- **Weizenprodukte**

Allerdings reagieren nur wenige Menschen mit einem RDS auf ein bestimmtes Nahrungsmittel.

Laktoseintoleranz

Laktose (Milchzucker) ist ein Zucker, den man in Milch und vielen verarbeiteten Lebensmitteln findet. Es gibt die sogenannte Laktoseintoleranz, die sehr ähnliche Symptome hat wie das RDS. Laktoseintoleranz bedeutet, dass der Körper Laktose nicht verdauen kann. Um Laktose zu verdauen, muss der Körper ein Enzym namens Laktase produzieren. Laktase wird normalerweise von Zellen im Dünndarm produziert. Laktase zerkleinert Milchzucker (Laktose) in einfachere Formen, die dann ins Blut aufgenommen werden können. Wenn es nicht genug Laktase gibt, erreicht der Milchzucker den Dickdarm, wo er von Bakterien zu kurzkettigen Fettsäuren, Kohlendioxid und Wasserstoff umgewandelt wird. Das führt zu:

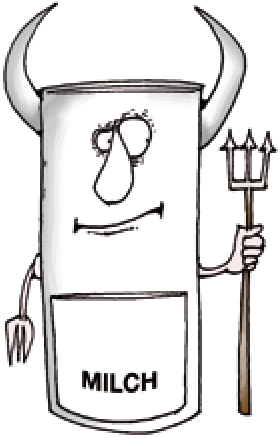

- Übelkeit
- Krämpfen
- einem Blähbauch
- Blähungen und
- Durchfall

Die Symptome beginnen ungefähr 30 Minuten bis zwei Stunden nachdem man Nahrungsmittel zu sich genommen hat, die Laktose enthalten. Wie schlimm die Symptome sind, hängt davon ab, wie viel Laktose ein Mensch verträgt.
In manchen Studien hat man bei bis zu einem von fünf Menschen mit einem RDS, die ins Krankenhaus gingen, eine verminderte Laktosetoleranz festgestellt. Es gibt einfache Untersuchungsmethoden, die zeigen, ob nicht genug Laktase im Körper vorhanden ist (Laktasemangel).
Laktoseintoleranz wird oft mit dem Erwachsenwerden schlimmer. Das kommt daher, dass weniger von dem Enzym Laktase im Darm produziert wird. Laktoseintoleranz betrifft manche Bevölkerungsgruppen mehr als andere. Fast 100% der Menschen aus Südostasien haben eine Laktoseintoleranz, aber weniger als 5% der Menschen in Nordwesteuropa sind laktoseintolerant.
Es gibt viele Menschen mit Laktasemangel, die keine Probleme mit Milchprodukten haben, und andere mit normaler Laktase, die keine Milchprodukte vertragen.

Für Menschen, die eine Laktoseintoleranz haben und die keine Milch oder Milchprodukte vertragen, kann es hilfreich sein, Nahrungsmittel wegzulassen, die Laktose enthalten, oder diese Nahrungsmittel stark zu reduzieren. Wenn Sie denken, dass Sie ein Problem durch eine Laktoseintoleranz haben, lesen Sie bitte den Teil zur Ernährung in Kapitel 3.
Es ist allerdings sehr selten, dass jemand Laktose komplett meiden muss.

Fruktosemalabsorption
Fruktose (Fruchtzucker) ist ein weiterer Zucker, der Probleme verursachen kann. Man findet Fruktose vor allem in Obst, Mais, Rosinen, Honig, Kartoffeln, Fruchtsäften, Softdrinks und Süßstoffen. Absorbieren bedeutet aufnehmen, bei einer Fruktosemalabsorption kann Fruktose also nicht richtig aus dem Darm ins Blut aufgenommen werden, weil ein Fruktosetransporter, der dafür zuständig ist, überfordert ist. Die Fruktose wird dann von Bakterien umgewandelt und die Symptome sind die gleichen wie bei einer Laktoseintoleranz. Sehr große Mengen an Fruktose können auch bei gesunden Menschen Probleme bereiten, weil der Fruktosetransporter auch dann überfordert sein kann.
15-25% der Bevölkerung haben eine Fruktosemalabsorption und sie kommt häufig zusammen mit einem RDS vor. Es gibt einfache Tests, mit denen man eine Fruktosemalabsorption feststellen kann, zum Beispiel den sogenannten H2-Atemtest.

Menschen, die eine Fruktosemalabsorption haben, sollten weniger als 10g Fruktose pro Tag zu sich nehmen. Man kann auch für jeden Menschen einzeln feststellen, wie viel Fruktose er verträgt. Glukose (Traubenzucker), die unter anderem in Rohr- oder Rübenzucker enthalten ist, hilft bei der Fruktose-Aufnahme. Bananen enthalten viel Glukose und werden daher oft besser vertragen als Äpfel, die wesentlich mehr Fruktose enthalten. Fruktose wird auch besser zusammen mit eiweißreichen Mahlzeiten aufgenommen. Es sollte auch auf Alkohol und Zuckeralkohole wie Sorbit, die oft als Süßstoffe verwendet werden, verzichtet werden. Eine richtige Ernährungsumstellung führt meist zum Verschwinden der Symptome.

! ACHTUNG ! Verwechseln Sie die Fruktosemalabsorption nicht mit der Fruktoseintoleranz. Die Fruktoseintoleranz ist eine erbliche Stoff-

wechselkrankheit, die sehr selten ist, sich schon im Säuglingsalter zeigt, und mit anderen Symptomen einhergeht.

Sorbitmalabsorption

Sorbit ist ein Zuckeralkohol, der zum Beispiel in getrocknetem Obst enthalten ist. Es wird häufig als Süßungsmittel eingesetzt und kommt oft in Diabetikerkost, Kaugummi, Bonbons und anderen Lebensmitteln vor, die als „zuckerfrei" gekennzeichnet sind.
Sorbit hemmt den Fruktosetransporter im Darm und kann deshalb eine Fruktosemalabsorption verschlimmern. Manchmal kann Sorbit auch alleine zu Problemen wie bei einer Laktoseintoleranz oder Fruktosemalabsorption führen.

Wenn Sie eine Fruktosemalabsorption haben, oder vermuten, dass Ihre Symptome mit Sorbit zusammenhängen könnten, sollten Sie sorbithaltige Lebensmittel meiden.

Glutenunverträglichkeit

Damit ist eigentlich eine Unverträglichkeit gegen die einzelnen Bestandteile des Glutens gemeint, so genannte Gliadine oder auch Klebeeiweiße, die in vielen Getreidesorten vorkommen. Durch eine Überempflindlichkeit der Dünndarmschleimhaut entsteht ein chronischer Entzündungsreiz, durch den die innere Auskleidung der Darmschleimhaut beschädigt werden kann. Dadurch können Nährstoffe nur schlecht aufgenommen werden und verbleiben unverdaut im Darm. Die Symptome und die Ausprägung des Krankheitsbildes können sehr unterschiedlich sein, was das Erkennen erschwert.
Mögliche Symptome sind Gewichtsverlust, Durchfall, Erbrechen, Appetitlosigkeit, Müdigkeit, Malabsorption (verminderte Nährstoffaufnahme), und im Kindesalter eine Gedeihstörung (verlangsamte körperliche Entwicklung). Für die Testung werden spezielle Antikörper im Blut bestimmt und im Rahmen einer Magenspiegelung Proben aus der Dünndarmschleimhaut (Biopsien) entnommen und feingeweblich unter dem Mikroskop untersucht. Die Behandlung der Zöliakie besteht derzeit fast ausschließlich aus einer lebenslangen glutenfreien Diät (z.B. Verzicht auf Kartoffeln, Mais, Reis, Hirse, Sojabohnen, Produkte aus Weizen, Gerste, Roggen etc.).
Falls diese Maßnahmen nicht ausreichen, kann in Ausnahmefällen mit immunsuppressiven Medikamenten behandelt werden.

Ballaststoffe in Ihrer Nahrung

Ballaststoffe in der Ernährung sind für jeden wichtig. Man findet sie in Nahrungsmitteln wie Frühstücksflocken aus Vollkorn und Vollkornbrot, Obst und Gemüse. Es gibt unterschiedliche Arten von Ballaststoffen und es ist wichtig, verschiedene Nahrungsmittel zu essen, die natürlicherweise viele Ballaststoffe enthalten, weil diese Nahrungsmittel uns auch mit Vitaminen, Mineralstoffen und Nährstoffen versorgen. Obst und Gemüse enthalten Stoffe wie „Bioflavinoide", denen man eine schützende Wirkung vor Krebs zuschreibt.

Ballaststoffe kommen aus den Pflanzen, die wir essen. In Pflanzen stecken unterschiedliche Mengen an löslichen und unlöslichen Ballaststoffen. Wir brauchen beide Arten von Ballaststoffen für eine gesunde Ernährung. Unlösliche Ballaststoffe werden nicht vom Körper verdaut; sie helfen anderen Nahrungsmittelresten, durch den Darm zu wandern, halten den Darm gesund und helfen gegen Verstopfung. Gute Quellen für unlösliche Ballaststoffe sind: Frühstücksflocken aus Vollkorn, Vollkornbrot und Gemüse mit Schale. Lösliche Ballaststoffe werden zum Teil im Darm verdaut und es gibt Anzeichen dafür, dass sie helfen, das Cholesterin im Blut zu senken. Gute Quellen für lösliche Ballaststoffe sind: Haferflocken, Obst, Gemüse und Hülsenfrüchte (Bohnen und Linsen).

Die meisten Hausärzte halten ihre RDS-Patienten dazu an, mehr Ballaststoffe zu essen, weil Ballaststoffe tatsächlich gegen Verstopfung helfen. In der westlichen Welt ist die Ernährung meist ballaststoffarm. Es wurden einige Studien durchgeführt, die untersucht haben, was mit Menschen mit einem RDS passiert, wenn sie beginnen, mehr Ballaststoffe in Form von Kleie zu essen. Diese Studien zeigen, dass Kleie Menschen mit Durchfall nicht hilft, die Reizdarmsymptome

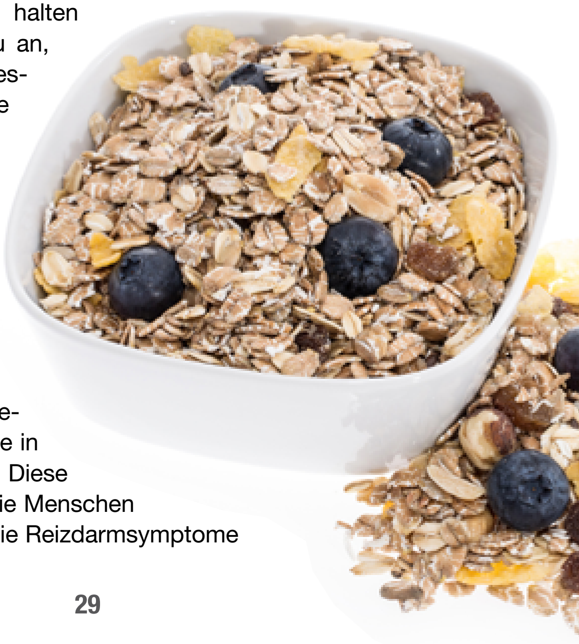

© HandmadePictures/fotolia.com

zu lindern, und dass sie manche Symptome verschlimmern kann. Denken Sie daran, dass Kleie nur eine von vielen Ballaststoffquellen ist. Wenn Sie ein RDS mit Verstopfung haben, ist es wahrscheinlicher, dass Sie Ballaststoffe als hilfreich empfinden.
Mehr Ballaststoffe zu Ihrer Diät hinzuzufügen kann manche Probleme kurzzeitig verschlimmern, solange sich Ihr Darm an die Veränderung gewöhnt. Sie sollten die Ballaststoffmenge langsam über mehrere Tage hinweg erhöhen. Wenn Sie mehr Ballaststoffe essen, kann es sein, dass Sie stärkere Blähungen bekommen. Wenn Sie glauben, dass Sie Probleme mit Ballaststoffen haben, kann es sich lohnen, unterschiedliche Arten von Ballaststoffen auszuprobieren, um zu sehen, welches Produkt am besten zu Ihnen passt. Vollkornprodukte finden Sie in Supermärkten oder im Reformhaus. Suchen Sie die heraus, die nicht auf Weizen oder Kleie basieren.

Medizinische Untersuchungen

Die meisten Menschen mit einem RDS brauchen gar keine technischen Untersuchungen. Es ist möglich, ein RDS aufgrund Ihrer Symptome zu diagnostizieren. Das RDS ist sehr häufig, und wenn Sie die folgenden Probleme haben, ist es sehr unwahrscheinlich, dass Ihnen irgendetwas anderes fehlt:

- **Symptome, die kommen und gehen**
- **Bauchschmerzen, die besser werden, wenn Sie Stuhlgang haben oder**
- **Bauchschmerzen im Zusammenhang mit Verstopfung oder Durchfall**

Hinzukommen können eines oder mehrere der folgenden Symptome:

- **eine Veränderung in der Häufigkeit, mit der Sie Stuhlgang haben**
- **eine Veränderung der Art Ihres Stuhls – hart oder lose**
- **Anstrengung, um Stuhlgang haben zu können**
- **das Gefühl, dringend Stuhlgang haben zu müssen**
- **das Gefühl, dass Sie den Darm nicht komplett entleert haben**
- **das Ausscheiden von Schleim oder**
- **ein aufgeblähter oder geschwollener Bauch**

Wenn Sie einen Arzt aufsuchen müssen, wird dieser Sie nach Ihrer Ernährung und Ihren Medikamenten fragen. Vielleicht will er auch etwas über Ihren Lebensstil, Ihre Arbeit und besonders mit Stress verbunde-

nen Ereignisse in Ihrem Leben wissen. Es könnte Ihnen helfen, sich ein paar Notizen zu machen, die Sie mit zum Arzt nehmen.

Ernstere Probleme

Es kann sein, dass Sie mehr Untersuchungen brauchen, falls Sie:

- Blut mit Ihrem Stuhl ausscheiden
- Gewicht verlieren
- nachts wegen Schmerzen oder Durchfall aufwachen oder
- über 40 sind, wenn Sie das erste Mal Veränderungen Ihres Stuhlgangs bemerken.

Wenn Ihre Hauptbeschwerde Durchfall ist, werden Sie Ihrem Arzt eine Stuhlprobe geben müssen, um sicher zu gehen, dass Sie keine Infektion haben.
Die meisten Fälle von rektalen Blutungen sind nicht ernst und kommen oft von geschädigten Darmwänden. Falls Sie sich beim Stuhlgang anstrengen müssen und harten Stuhl ausscheiden, kann es sein, dass die Auskleidung Ihres Darms beschädigt wird und blutet. In diesem Fall ist das Blut hellrot (wie bei Nasenbluten). Oder die Blutung kann bedeuten, dass Sie vergrößerte Hämorrhoiden haben.

! Es ist wichtig, dass Ihr Arzt sicherstellt, dass die Blutung kein Zeichen für etwas Ernsteres ist.

Wenn Sie aus dem Rektum bluten, sollten Sie das nicht ignorieren, weil es ein Anzeichen für Krebs oder eine andere gefährliche Darmerkrankung sein kann.
Wenn Sie Symptome haben, von denen Sie oder Ihr Arzt denken, dass sie eine ernstere Ursache haben, sollte Ihr Hausarzt Sie an einen Gastroenterologen überweisen. Ihr Arzt wird sicher gehen wollen, dass Sie keinen Darmkrebs oder andere Darmerkrankungen haben.

Denken Sie daran …

… Es gibt viele Theorien darüber, was das RDS auslöst. Die Kenntnisse hierzu könnten Ihnen bei Ihren Überlegungen helfen, wie Sie mit Ihren Symptomen besser umgehen können. Es wurde bisher aber keine alleinige Ursache für das RDS gefunden.

… Untersuchungen bei Menschen mit einem RDS zeigen, dass der Darm normal und gesund aussieht.

… Menschen mit einem RDS haben einen empfindlichen Darm, der nicht richtig funktioniert.

… Eine gesunde Ernährung ist wichtig, damit der Darm richtig funktioniert.

… Die meisten Menschen mit einem RDS brauchen keine medizinisch-technischen Untersuchungen.

… Wenn Sie Blut mit dem Stuhl ausscheiden, Gewicht verlieren oder nachts von Schmerzen geweckt werden, sind eingehende Untersuchungen nötig.

Kapitel 3

Was Sie tun können, um sich selbst zu helfen

In diesem Kapitel geht es darum, wie Sie besser mit Ihren Reizdarmbeschwerden umgehen können.

Übernehmen Sie die Kontrolle über Ihre Ernährung

Jeder weiß, dass man sich mit bestimmten Nahrungsmitteln den Magen verderben kann, und dass das von Mensch zu Mensch unterschiedlich ist. Es gibt viele Theorien darüber, wie das, was wir essen, mit dem RDS zusammenhängen könnte, aber es gibt sehr wenige wissenschaftliche Erkenntnisse als Grundlage dieser Theorien. Reaktionen auf Essen hängen von der Person ab. Echte Nahrungsmittelallergien sind sehr selten, aber manche Menschen vertragen bestimmte Nahrungsmittel nicht. Viele klagen über Verdauungsstörungen, nachdem sie bestimmte Nahrungsmittel gegessen haben (zum Beispiel Sellerie, Zwiebeln, Knoblauch, Gurken und schwerverdauliches Essen) und vermeiden diese eher.

Die moderne Ernährung

Manche Menschen denken, dass unsere moderne Ernährung das RDS verursachen könnte. In den vergangenen 50 Jahren gab es große Veränderungen in den Ernährungsgewohnheiten der Menschen.

- Wir essen weniger stärkehaltige Nahrungsmittel wie Brot, Hafergrütze und Kartoffeln.
- Wir essen mehr raffinierte Zucker und Fett.
- Mahlzeiten finden wegen unseres hektischen Lebensstils oft unter Zeitdruck oder gar nicht statt.
- Die Verwendung von chemischen Zusatzstoffen im Essen ist dramatisch gestiegen.

Die Ernährung ist die wichtigste Veränderung, die wir in Bezug auf das Milieu unseres Verdauungstrakts vornehmen können. Heutzutage erkennen viele Menschen an, wie wichtig eine gesunde Ernährung ist, weil sie festgestellt haben, dass sie sich durch eine gesunde Ernährung besser fühlen. Es gibt immer mehr Naturkost- und Bioläden, und die Menschen essen insgesamt bewusster. Eine gesunde Ernährung bedeutet, viel frisches Obst und Gemüse zu essen und Nahrungsmittel zu sich zu nehmen, die wenig Fett und Salz und viele Ballaststoffe enthalten.

„An Tagen, an denen ich Schmerzen habe, steige ich auf die ballaststoffreichen Frühstücksflocken um, und es wird wirklich besser, das hilft mir.“

Nahrungsmittelunverträglichkeiten

Menschen mit einem RDS stellen häufig fest, dass bestimmte Nahrungsmittel Symptome hervorrufen. Die Nahrungsmittel, bei denen am häufigsten Probleme festgestellt werden, sind Milchprodukte und Weizenprodukte. Ein Weg, herauszufinden, ob Nahrungsmittel das RDS verursachen, ist, diese Nahrungsmittel wegzulassen. In der Forschung hat man herausgefunden, dass Ausschlussdiäten bei manchen Menschen die Reizdarmsymptome beseitigen können. Es ist wichtig, nicht so viele Nahrungsmittel wegzulassen, dass Sie sich mangelernähren und krank werden.

Ausschlussdiäten sind mühsam. Sie sollten so eine Diät nicht auf eigene Faust beginnen, weil diese Diäten kompliziert sind und schädlich sein können, wenn sie nicht richtig gemacht werden. Am besten ist es, wenn Ihr Hausarzt die Diät mit Ihnen überwacht. Ausschlussdiäten bestehen aus Nahrungsmitteln, von denen bekannt ist, dass sie keine negativen Reaktionen hervorrufen. Man muss sich mindestens zwei Wochen lang streng an die Diät halten, damit sie funktioniert. Wenn Ihre Symptome nach zwei Wochen verschwunden sind, können Sie beginnen, Ihrer Diät wieder Nahrungsmittel hinzuzufügen, immer nur eines auf einmal. Es sollte Ihnen möglich sein, herauszufinden, welche Nahrungsmittel Ihre Symptome verursachen. Wenn Ihre Symptome nach zwei Wochen noch nicht weg sind, haben Sie keine Nahrungsmittelunverträglichkeit. Wenn Sie diese strenge Diät ausprobieren wollen, sollten Sie das mit Ihrem Arzt besprechen.

Es kann sein, dass Sie schon wissen, welche Art von Nahrungsmitteln Ihre Symptome hervorrufen. Wenn Ihre Reizdarmsymptome nicht zu schlimm sind, können Sie alleine herausfinden, ob bestimmte Nahrungsmittel Probleme verursachen. Denken Sie daran, immer nur eine Art von Nahrungsmitteln auf einmal wegzulassen. Benutzen Sie ein Ernährungstagebuch, um sich leichter daran zu erinnern, was Sie in den 24 Stunden vor einem schweren Anfall gegessen haben.

„Anscheinend bringt Milch mich ganz schön durcheinander, und ich liebe Milch.“

Beispiele von Nahrungsmitteln, die besonders wahrscheinlich ein RDS verursachen

Die Symptome, die Sie haben, könnten von einer Laktoseintoleranz verursacht sein. Sie können das herausfinden, indem Sie einfach Milchprodukte weglassen. Wenn Sie eine Laktoseintoleranz haben, werden Ihre Symptome verschwinden, wenn Sie das tun.

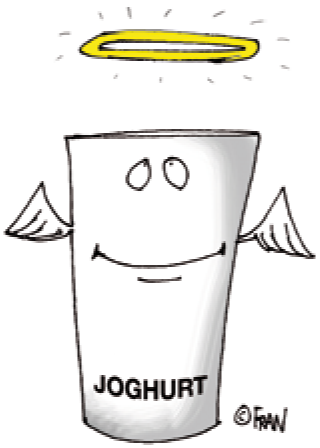

Milchprodukte:

- Milch
- Sahne
- Butter
- Quark
- Eis
- Käse
- Frischkäse

Joghurt ist ein Milchprodukt, das nur geringe Mengen an Laktose enthält. Die Bakterien, die aus Milch Joghurt machen, wandeln einen Teil der Laktose in Milchsäure um. Wenn Sie eine Laktoseintoleranz haben, kann es sein, dass Sie Joghurt essen können, ohne Reizdarm-symptome zu bekommen.

Verarbeitete Lebensmittel enthalten häufig Laktose-Zusätze. Sie sollten die Liste der Inhaltsstoffe überprüfen.

Beispiele für verarbeitete Lebensmittel:

- Brot und andere Backwaren
- Verarbeitete Frühstücksflocken
- Fertig-Kartoffelbrei, Fertigsuppen und Frühstücksgetränke
- Margarine
- Fleisch-Aufschnitt
- Salatsaucen
- Süßigkeiten und andere Snacks
- Backmischungen für Pfannkuchen und Kekse

Manche Nahrungsmittel, die als „milchfrei“ ausgewiesen sind, wie zum Beispiel Kaffeeweißer-Pulver und Glasuren, können dennoch Zutaten enthalten, die aus Milch gewonnen werden und Laktose enthalten.
Es kann sein, dass Sie Milchprodukte nicht komplett weglassen müssen. Es könnte ausreichen, nur den Milchkonsum zu reduzieren, um Ihre Symptome zu vermeiden. Ihr Körper sollte fähig sein, kleine Mengen Laktose problemlos zu verdauen. Es könnte helfen, gleichzeitig etwas anderes zu essen, wenn Sie ein Milchprodukt zu sich nehmen.
Manchen Menschen helfen auch Laktase-Tabletten, die man vor dem Essen einnehmen kann. So kann man die Laktase, die bei einer Laktoseintoleranz vermindert vorhanden ist, ersetzen. Laktase-Tabletten sind frei verkäuflich, man bekommt sie in manchen Super- und Drogeriemärkten oder im Reformhaus.
Wenn Sie eine reduzierte Laktosetoleranz haben und weniger laktosehaltige Nahrungsmittel zu sich nehmen, werden Ihre Symptome komplett verschwinden.

Denken Sie daran, dass Milchprodukte eine wichtige Kalziumquelle sind, welches man lebenslang für das Wachstum, die Instandhaltung und die Reparatur der Knochen braucht.

Jeder braucht Kalzium, besonders Kinder im Wachstum und Frauen nach den Wechseljahren, um Osteoporose (Knochenschwund) vorzubeugen. Käse und Joghurt sind gute Kalziumquellen und es kann sein, dass Sie diese essen können, selbst wenn Sie keine Milch vertragen. Milch und die meisten Milchprodukte gibt es auch laktosefrei, achten Sie auf die Kennzeichnung auf der Verpackung.

Andere gute Kalziumquellen:
- Fisch mit weichen Gräten, die man essen kann (wie zum Beispiel Lachs und Sardinen);
- Soja-Milch, die mit Kalzium angereichert wurde. Man bekommt sie in Supermärkten und im Reformhaus.

Wenn Sie all diese Nahrungsmittel meiden, können Sie sich von Ihrem Hausarzt allgemein zum Thema Kalzium-Ersatz beraten lassen. Wenn Sie sich große Sorgen machen, kann ein Ernährungsberater Ihre Diät überprüfen und Ihnen sagen, ob Sie Kalzium-Ersatzmittel brauchen.

Weizenprodukte

Manche Menschen mit einem RDS haben Probleme mit dem Essen von Weizenprodukten. Das kommt normalerweise daher, dass Weizenprodukte recht schwer verdaulich sind.

Beispiele für Weizenprodukte:
- Brot
- Gebäck
- Frühstücksflocken auf Grundlage von Weizen (auf Hafer basierende Frühstücksflocken sollten keine Probleme verursachen).

Künstliche Süßstoffe

Man hat herausgefunden, dass künstliche Süßstoffe und manche Zucker Reizdarmsymptome verursachen, zum Beispiel:
- Sorbit
- Fruktose

Sorbit ist eigentlich ein Abführmittel, das Durchfall auch bei Menschen verursacht, die keine Verdauungsprobleme haben.

Getränke

Es gibt Hinweise darauf, dass Kaffee und Alkohol Probleme verursachen können, weil sie dehydrierend (dem Körper Wasser entziehend) wirken können. Wenn im Verdauungssystem nicht genug Flüssigkeit vorhanden ist, kann man Probleme beim Verdauen des Essens haben, und die Verstopfung wird schlimmer.

Obst und Gemüse

Menschen mit einem RDS sagen oft, dass sie Probleme haben, nachdem sie Obst und Gemüse gegessen haben. Es gilt als gut für eine gesunde Ernährung, pro Tag mindestens fünf Portionen Obst oder Gemüse zu essen. Ein Glas mit purem Obstsaft gilt als eine Portion Obst. Manche Menschen, die ein RDS mit Durchfall haben, haben Probleme, wenn sie so viel Obst und Gemüse essen. Sie müssen herausfinden, was zu Ihnen passt. Menschen mit Verstopfung sollten merken, dass ihre Symptome besser werden, wenn sie mehr Obst und Gemüse essen.
Wenn Ihre Symptome durch Obst und Gemüse schlimmer werden, könnte es auch sein, dass Sie eine Fruktosemalabsorption haben.

Nahrungsmittelallergien

Es ist sehr unwahrscheinlich, dass Ihr RDS aufgrund einer Nahrungsmittelallergie besteht. Viele Menschen denken, sie seien auf ein bestimmtes Lebensmittel allergisch, weil es bei ihnen Unwohlsein verursacht. Das ist wahrscheinlich keine Allergie, sondern eine Unverträglichkeit gegenüber diesem Lebensmittel, die sehr unangenehme Symptome verursachen kann, aber nicht gefährlich ist. In der Medizin gilt es als allergische Reaktion, wenn das Immunsystem mit etwas reagiert und dadurch Symptome wie zum Beispiel Hautausschlag, ein Anschwellen des Gesichts oder Atemnot verursacht. Das muss man ernst nehmen, es ist lebensgefährlich. Beispiele sind eine Erdnuss- oder Erdbeerallergie. Nur sehr wenige Menschen haben echte Nahrungsmittelallergien.

Allergie- und Unverträglichkeitstests

Bei einem Esstest bekommt man eine kleine Portion eines Lebensmittels zu essen, von dem man denkt, dass es eine Reaktion verursachen könnte. Das Nahrungsmittel kann in einer Kapsel versteckt sein, so dass man nicht weiß, was man gegessen hat.

Pricktests an der Haut und Blutuntersuchungen werden manchmal gemacht, um zu sehen, ob man allergisch gegen Nahrungsmittelbestandteile ist. Diese Tests sind nicht verlässlich, weil sie oft eine positive Reaktion auf Nahrungsmittel zeigen, die keine Probleme verursachen, und weil sie echte Allergien manchmal nicht anzeigen.

! ACHTUNG ! Es gibt viele Unverträglichkeitstests, die nicht auf guten wissenschaftlichen Grundlagen beruhen. Leider wird gelegentlich versucht, mit den Symptomen der Menschen Geld zu verdienen.

Probleme mit Blähungen

Wie schon erwähnt, berichten Menschen mit einem RDS oft von Problemen durch Blähungen. Sie müssen aufstoßen, rülpsen oder haben ein Blähgefühl, laute Bauchgeräusche und abgehende Winde. Diese Symptome können alle sehr unangenehm sein.

Durch bestimmte Nahrungsmittel wird viel Gas gebildet. Zum Beispiel:
- Bohnen
- Kohl und Rosenkohl

- Linsen
- Äpfel und Apfelsaft
- Trauben

Um die Luftmenge im Magen zu reduzieren, vermeiden Sie:
- Kohlensäurehaltige Getränke
- Kaugummi
- hastiges essen

> *„Ich liebe Sprudelwasser, ich habe entdeckt, dass ich nur einen Tropfen Zitronensaft rein tun muss, das nimmt das Zischen weg."*

Gärungsprozesse im Darm

In letzter Zeit fragt man sich, welche Bakterienarten und -menge im Darm wichtig sind. Im menschlichen Darm befinden sich mehr als 400 Bakterienarten, und Forscher wissen noch nicht genau, wie wichtig sie für unsere Gesundheit sind. Wir brauchen Bakterien im Dickdarm, um Nahrungsmittelprodukte zu vergären. Manche Menschen glauben, dass dieser Vorgang bei Menschen mit einem RDS nicht richtig funktioniert. Sie vermuten, dass ein Auslöser, vielleicht eine Infektion oder ein Antibiotikum, die Art von Bakterien im Darm verändert hat. Wenn die normalen Darmbakterien nicht vorhanden sind, können andere Bakterien und Hefen ihren Platz einnehmen. Hefen vergären Essen anders als normale Darmbakterien und es heißt, dass dieser Unterschied bei manchen Menschen die Reizdarmsymptome hervorrufen kann. Es gibt noch nicht genug Forschungsergebnisse, um zu wissen, ob das eine wesentliche Ursache des RDS ist.

Probiotika sind Nahrungsmittel, die lebende Bakterien, wie zum Beispiel Lactobacillus acidophilus und Bifobacterium bifidum, enthalten, von denen es heißt, dass sie sich positiv auf die Gesundheit auswirken. Lebende Bakterien findet man in Joghurt mit lebenden Kulturen, zum Beispiel Biojoghurt, und Sauermilch-Getränken. Wesentlich höhere Konzentrationen an Probiotika kann man in speziell hergestellten Gesundheits-Lebensmitteln oder in speziellen Medikamenten finden. Es gibt Forschungsergebnisse, die zeigen, dass diese höher konzentrierten Zubereitungen bei manchen Reizdarmsymptomen wie einem Blähbauch oder Blähungen helfen könnten.

„Ich habe angefangen, Yakult zu nehmen. Ich kann nicht sagen, dass es mir besser geht, aber ich fühle mich nicht so unbehaglich, ich kann anscheinend besser damit umgehen."

! ACHTUNG ! Es gibt viele Gesundheits-Lebensmittel, deren Nutzen nicht wissenschaftlich erwiesen ist. Leider wird oft versucht, mit den Symptomen der Menschen Geld zu verdienen.

Ihre Ernährung

Wenn Sie denken, dass Ihr RDS durch Ihre Nahrung verursacht werden könnte, können Sie versuchen, Ihre Ernährung zu ändern. Man wird leicht ganz besessen von der Ernährung, wenn man ein RDS hat, und wenn man nicht vorsichtig ist, kann man in die Falle tappen viele Nahrungsmittel wegzulassen, auch wenn das gar nicht nötig ist. Wenn Sie aufgehört haben, eine bestimmte Sorte von Lebensmitteln zu essen, weil Sie denken, dass sie bei Ihnen Probleme hervorruft, lohnt es sich, es damit später nochmal zu versuchen. Sie müssen ein Detektiv bezüglich Ihrer Ernährung sein, weil die Reaktionen auf Nahrungsmittel bei jedem so anders sind. Was bei dem einen funktioniert, macht es bei jemand anderem vielleicht schlimmer. Denken Sie daran, dass ein regelmäßiges Essverhalten hilft.

Manche Lebensmittel helfen manchen Menschen...

„Ich habe festgestellt, dass ich Probleme habe, wenn ich kein Obst esse."

„Ich esse jeden Morgen eine Banane, ich muss nicht mehr auf die Toilette rennen wie früher."

„Ich esse viel grünes Gemüse aus dem Wok. Ich habe das Gefühl, dass ich viel langsamer esse, wenn ich Wok-Gemüse esse, ich kaue richtig."

„Ich habe herausgefunden, dass mir Haferbrei hilft."

„Wenn ich mich richtig schlecht fühle, esse ich einfach ein bisschen Suppe, das macht es ein bisschen besser."

„Wenn es mir schlecht geht, esse ich weißen gekochten Reis mit Hühnergeschnetzeltem.“

… aber verschlimmern es bei anderen.

„Ein hoher Ballaststoffgehalt hat Durchfall verursacht, den ich nicht kontrollieren konnte. Ich bin mit Windeln herumgelaufen, weil ich es einfach nicht kontrollieren konnte, nachdem ich Kleie-Flocken gegessen hatte.“

„Ich kann Bananen nicht anrühren, die rutschen einfach direkt durch mich durch.“

„Orangen haben einen ziemlich schlechten Einfluss, frische Orangen.“

„Kirschtomaten, ich habe sie auf einem belegten Brot gegessen und am Sonntag Morgen war ich völlig fertig von den Blähungen, dem ständigen auf die Toilette rennen, und davon, mich mit Stuhl schmutzig zu machen.“

„Wenn ich Sprossen esse, ohhh je, dann kriege ich schmerzhafte Blähungen.“

„Ich lasse Sachen mit Stückchen drin weg, wie Körnerbrot und Erdnussbutter mit Erdnussstückchen. Das erleichtert es ein bisschen, ich habe mich gefragt, ob die Stückchen es schlimmer gemacht haben.“

„Ich glaube, bei dem Bedürfnis auf die Toilette zu gehen hat es mir geholfen, mich von fettigen Lebensmitteln fernzuhalten.“

„Ich glaube, scharfes Essen ist noch etwas, das es auslösen kann. Wie Curry und so.“

Wie man isst

Manche Patienten haben herausgefunden, dass die Art, wie sie essen, zu Reizdarmsymptomen führt. Schnell zu essen und Luft mit herunterzuschlucken kann zu Problemen führen.

> ***„Es liegt mehr an meinem Essverhalten als daran, was ich wirklich esse. Wenn ich das Essen hinunterschlinge hilft das nicht gerade, und wenn ich eine große Mahlzeit anstatt vieler kleiner Mahlzeiten esse, ist das nicht unbedingt hilfreich."***

> ***„Wenn ich mich vollstopfe, dann kriege ich Blähungen."***

Bewegung

Wir wissen, dass Bewegung nicht nur den Körper fit hält, sondern auch das Wohlbefinden steigert. Bewegung kann für Menschen mit einem RDS besonders hilfreich sein, weil:

- Bewegung gegen Verstopfung hilft
- Bewegung dazu führt, dass man sich mit sich selbst wohler fühlt
- Bewegung dabei hilft, ein gesundes Körpergewicht zu halten und
- Bewegung den Appetit mindert.

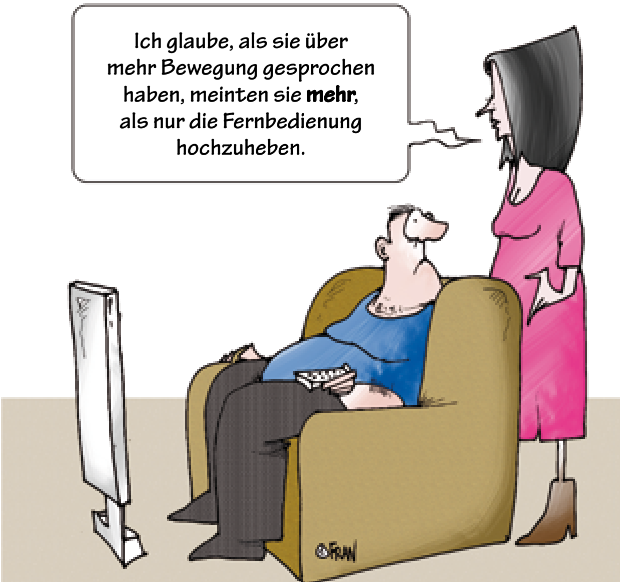

Hier sind einige Tipps zur Bewegung

- Probieren Sie eine Art der Bewegung aus, die die meisten großen Muskeln des Körpers in Anspruch nimmt, zum Beispiel Walken, Laufen oder Schwimmen.
- Fangen Sie langsam an, sich mehr zu bewegen, sowohl in Hinsicht auf die Mühe als auch auf die Zeit, die Sie investieren. Sie sollten die Bewegung in kleinen Stufen und über mehrere Wochen hinweg steigern.
- Die Bewegung sollte Sie ins Schwitzen bringen und Sie sollten eine Zeit lang außer Atem kommen, aber Sie sollten währenddessen noch reden können.
- Sie sollten sich mindestens 20 Minuten lang dreimal die Woche bewegen.

„Es ist schwer, Bauchübungen zu machen, wenn man richtig aufgebläht ist und es wehtut, aber Sit-ups und andere Bauchübungen bringen die Sache in Bewegung, dann fängt es an zu arbeiten."

Wie man mit Stress umgehen kann

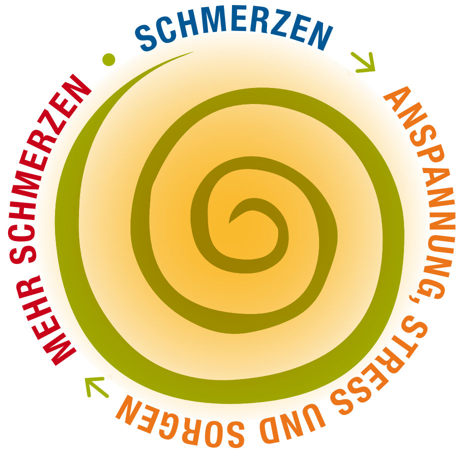

Entspannung

Zu lernen, wie man sich entspannt, kann für Menschen mit einem RDS aus zwei Gründen hilfreich sein:

- Entspannung ist ein Weg, mit Stress umzugehen und ihn zu reduzieren und
- Entspannung kann ein Weg sein, wie Sie Schmerzen lindern können.

Durch Entspannung kann man womöglich den Teufelskreis durchbrechen:
Es gibt mehrere Arten, wie man sich selbst beibringen kann, sich zu entspannen. Man kann Entspannung üben und darin besser werden. Es ist also möglich, in einem Kurs oder von einem Therapeuten zu lernen, wie man sich entspannt. Viele Frauen lernen Entspannungstechniken in Geburtsvorbereitungskursen, wenn sie schwanger sind.

Stress

Von Stress heißt es oft, dass er zum RDS führt. Die meisten Menschen mit einem RDS sagen, dass sie ein belastendes Ereignis mit ihren Symptomen verbinden können. Manchmal kann Stress gut sein. Wir brauchen eine bestimmte Menge Stress, damit wir effektiv handeln und darüber nachdenken, was wir tun. Aber Stress kann auch schädlich werden, wenn wir nicht damit umgehen können, oder nicht wissen, wie wir mit der Ursache für den Stress umgehen sollen. Manche sind möglicherweise schneller gestresst als andere. Das sind Menschen, die:

- einen schlechten Gesundheitszustand,
- keine Arbeit,
- finanzielle Sorgen,
- einen komplizierten Lebens- und Arbeitsstil,
- keine effektiven Methoden, mit schwierigen Situationen umzugehen,
- emotionale Probleme,
- schlechte Vorerfahrungen oder
- ein geringes Selbstbewusstsein haben.

Wenn Sie denken, dass Ihr RDS mit Stress verbunden ist, gibt es Wege, mit dem Stress besser zurechtzukommen. Sie müssen vielleicht Veränderungen vornehmen an:

- Ihrem Lebensstil und Ihrer Arbeitsumgebung und
- Ihrem Verhalten und darin, wie Sie Ihren Körper benutzen.

Manchmal ist es nicht möglich, etwas an der Ursache für den Stress zu ändern, weil sie außerhalb der eigenen Kontrolle liegt. Man kann jedoch etwas tun, um die Auswirkungen des Stresses zu vermindern.

- Versuchen Sie, sich mindestens 20 Minuten dreimal die Woche zu bewegen. Bewegung ist beim RDS auch gut, weil sie gegen Verstopfung hilft. Sie können an einem Sportkurs teilnehmen oder abends einen strammen Spaziergang machen.
- Verbessern Sie Ihre Haltung im Stehen und Sitzen.
- Entwickeln Sie gesunde Lebensgewohnheiten.
- Hören Sie auf zu Rauchen und Alkohol zu trinken, oder versuchen Sie zumindest, die Anzahl der Zigaretten und die Menge, die Sie trinken, zu reduzieren.
- Sprechen Sie mit anderen, zum Beispiel mit Freunden oder der Familie, sie werden Sie vielleicht unterstützen.
- Hören Sie Musik und lesen Sie.
- Beginnen Sie mit entspannenden Hobbys.

Zu lernen, wie man den Körper entspannt, kann helfen, Stress zu reduzieren.
Entspannung kann auch helfen, Schmerzen zu lindern. Es gibt einfache Methoden, sich tagsüber während der Arbeit zu entspannen.

- Kurze, regelmäßige Pausen (jede Stunde fünf Minuten) sind entspannender als weniger längere Pausen.
- Kleine Gymnastikübungen helfen Menschen, die am Computer arbeiten, man kann zum Beispiel die Arm- und Beinmuskeln abwechselnd an- und entspannen.
- Atmen Sie ein paarmal tief ein und langsam aus, um eine direkte Stressreaktion oder Panikattacke zu stoppen.

Die erfolgreichsten Entspannungstechniken haben drei Komponenten.

1. Sie sollten sich auf ein Wort oder eine Bewegung konzentrieren, die Sie immer wiederholen.
2. Sie sollten vorbeiziehende Gedanken ignorieren.
3. Sie sollten Ihre Muskeln entspannen.

Entspannungstherapie hat sich in vielen Situationen als nützlich erwiesen und sollte den meisten Menschen mit einem RDS helfen. Versuchen Sie, sich eine halbe Stunde am Tag an einem Ort, an dem Sie

nicht gestört werden, zu entspannen. Leise, beruhigende Musik kann dabei helfen. Es gibt viele Entspannungs-CDs auf dem Markt.

> *„Ich hatte viele von diesen CDs und eine mit Farben, und eine mit Träumen, am Fluss langgehen und sowas, also... man probiert sie einfach mal aus, aber sie helfen einem, sich zu beruhigen."*

Progressive Muskelentspannung

Der folgende Abschnitt ist ein Beispiel für eine verbreitete Methode, die in Entspannungskursen unterrichtet wird. Bei dieser Methode lernt man, Muskelgruppen am Körper anzuspannen und locker zu lassen. Das Ziel ist es, die Muskeln zu entspannen. Feste muskuläre Anspannung führt im Anschluss zu tiefer Entspannung. Sie müssen sich der Gefühle bewusst werden, die Sie in Ihrem Körper spüren, wenn Sie Muskeln an- und entspannen. Es gibt 16 Muskelgruppen, die man an- und entspannen kann, und das ganze Programm dauert 20 bis 30 Minuten.

1. **Machen Sie mit Ihrer rechten Hand eine Faust, ohne den Oberarm zu benutzen.** Dann lassen Sie locker und denken darüber nach, wie sich Ihre Muskeln anfühlen.

2. **Drücken Sie Ihren rechten Ellbogen gegen die Stuhllehne nach unten, während Ihre Hand entspannt ist.** Dann lassen Sie locker und denken darüber nach, wie sich Ihre Muskeln anfühlen.

3. **Machen Sie mit Ihrer linken Hand eine Faust, ohne den Oberarm zu benutzen.** Dann lassen Sie locker und denken darüber nach, wie sich Ihre Muskeln anfühlen.

4. **Drücken Sie Ihren linken Ellbogen gegen die Stuhllehne nach unten, während Ihre Hand entspannt ist.** Dann lassen Sie locker und denken darüber nach, wie sich Ihre Muskeln anfühlen.

5. **Runzeln Sie die Stirn**. Dann lassen Sie locker und denken darüber nach, wie sich Ihre Muskeln anfühlen.

6. **Kneifen Sie die Augen zu und runzeln Sie die Nase.** Dann lassen Sie locker und denken darüber nach, wie sich Ihre Muskeln anfühlen.

7. **Beißen Sie die Zähne fest zusammen und ziehen Sie die Mundwinkel nach hinten.** Dann lassen Sie locker und denken darüber nach, wie sich Ihre Muskeln anfühlen.

8. **Ziehen Sie Ihr Kinn nach unten und drücken Sie Ihren Kopf nach Hinten gegen einen Widerstand, zum Beispiel die Rückenlehne Ihres Stuhls, spannen Sie Ihre Nackenmuskulatur an.** Dann lassen Sie locker und denken darüber nach, wie sich Ihre Muskeln anfühlen.

9. **Ziehen Sie Ihre Schultern nach hinten.** Dann lassen Sie locker und denken darüber nach, wie sich Ihre Muskeln anfühlen.

10. **Spannen Sie die Bauchmuskeln an (machen Sie Ihren Bauch hart).** Dann lassen Sie locker und denken darüber nach, wie sich Ihre Muskeln anfühlen.

11. **Spannen Sie den rechten Oberschenkel an (die Muskeln, die zum Knie ziehen).** Dann lassen Sie locker und denken darüber nach, wie sich Ihre Muskeln anfühlen.

12. **Strecken Sie Ihren rechten Fuß mit den Zehenspitzen nach unten.** Dann lassen Sie locker und denken darüber nach, wie sich Ihre Muskeln anfühlen.

13. **Ziehen Sie den rechten Fuß nach oben.** Dann lassen Sie locker und denken darüber nach, wie sich Ihre Muskeln anfühlen.

14. **Spannen Sie den linken Oberschenkel an (die Muskeln, die zum Knie ziehen).** Dann lassen Sie locker und denken darüber nach, wie sich Ihre Muskeln anfühlen.

15. **Strecken Sie Ihren linken Fuß mit den Zehenspitzen nach unten.** Dann lassen Sie locker und denken darüber nach, wie sich Ihre Muskeln anfühlen.

16. **Ziehen Sie den linken Fuß nach oben.** Dann lassen Sie locker und denken darüber nach, wie sich Ihre Muskeln anfühlen.

Sie sollten das zweimal am Tag 15 bis 20 Minuten lang üben. Wenn Sie das Gefühl haben, dass Sie das gut geübt haben und sich komplett entspannen können, können Sie das Ganze auf vier Bestandteile reduzieren.

1. **Beide Arme gleichzeitig**
2. **Gesicht und Kopf gleichzeitig**
3. **Hals und Oberkörper gleichzeitig**
4. **Beide Beine gleichzeitig**

Atmung und Entspannung

Atemübungen kann man überall machen. Eine ruhige Atmung ist langsam und wird mit entspannten Bauchmuskeln gemacht. Die Atmung bei Stress ist schneller, benutzt die Rippenmuskulatur, und die Schultern werden angespannt. Atemübungen können dann helfen, um wieder zu einer gesunden Atemfrequenz zu finden.

Entspannen Sie Ihren Kiefer, um langsam zu atmen
Lassen Sie Ihren Unterkiefer leicht nach unten fallen, als ob Sie ein wenig gähnen müssten. Lassen Sie Ihre Zunge ruhig am Mundboden ruhen. Lassen Sie Ihre Lippen weich werden. Atmen Sie langsam und in einem Dreier-Rhythmus: Einatmen – Ausatmen – Ruhe. Hören Sie auf, Wörter zu bilden, vielleicht vergessen Sie sogar ganz, an Wörter zu denken.

Wie man noch langsam atmen kann
Atmen Sie langsam tief ein. Dann atmen Sie langsam aus – spüren Sie nach, wie Sie langsam anfangen, sich zu entspannen. Spüren Sie, wie die Anspannung von Ihnen abfällt. Jetzt atmen Sie langsam in einem angenehmen Tempo weiter ein und aus.

Konzentrieren Sie sich auf Ihre Atmung. Zählen Sie im Kopf „Eins, zwei, drei“, während Sie einatmen. Oder wiederholen Sie jedes Mal, wenn Sie einatmen, ein bestimmtes Wort, wie „Frieden“ oder „Ruhe“ oder „Ich bin ganz ruhig und entspannt.“

Ein bisschen anders

Hören Sie auf Ihre Atmung. Stellen Sie sich vor, wie Ihre Anspannung ausgeatmet wird, bei jedem ausatmen ein bisschen mehr. Stellen Sie sich vor, dass Sie jedes Mal, wenn Sie einatmen, ein bisschen Ruhe und Frieden mit einatmen. Atmen Sie Spannung aus und Frieden ein, atmen Sie weich, fühlen Sie, wie der Frieden durch Ihren Körper fließt.

Denken Sie daran …

Sie können Ihr RDS selbst kontrollieren, indem Sie

… Ihre Ernährung verändern,
… sich mehr bewegen und
… etwas tun, um Ihren Stress zu reduzieren.

Kapitel 4

Wie man noch mit dem RDS umgehen kann

In diesem Kapitel erfahren Sie mehr über Behandlungsmöglichkeiten, die Sie ausprobieren können, für die Sie nicht extra zum Arzt gehen müssen.

Manchen Menschen haben alternative Therapien sehr geholfen, anderen nicht. Seien Sie daher kritisch, aber durchaus offen, wenn Sie diese ausprobieren. Wichtig ist auch, dass diese Verfahren Sie nicht zu viel Geld kosten. Im Zweifelsfall holen Sie sich Rat bei Ihrem Hausarzt.

Komplementäre und alternative Therapien

Komplementäre Therapien bzw. Naturheilverfahren haben das Ziel, den Menschen ganzheitlich zu behandeln. Menschen mit einem RDS sind manchmal enttäuscht, dass sie von der gewöhnlichen medizinischen Behandlung zu wenig Hilfe bekommen, so dass sie alternative Therapien ausprobieren wollen, um zu sehen, ob eine andere Herangehensweise bei ihnen funktioniert. Das kann teuer sein, aber viele Menschen finden komplementäre Therapien sehr hilfreich. Produkte aus dem Reformhaus oder solche, die Sie rezeptfrei in der Apotheke bekommen, können relativ günstig sein, aber es wird teurer, wenn Sie einen speziellen Therapeuten für seine Behandlung bezahlen. Manche Arztpraxen bieten ihren Patienten komplementäre oder alternative Therapien an. Viele komplementäre Therapien sind noch nicht so gut untersucht worden, wie man das bei Medikamenten und Behandlungsweisen der Schulmedizin macht. Die Schulmedizin kann meist nicht genau erklären, wie alternative Behandlungsweisen funktionieren.

! ACHTUNG ! Manche komplementäre Therapien basieren vielleicht nicht auf guten wissenschaftlichen Erkenntnissen. Leider wird gelegentlich versucht, mit den Beschwerden der Menschen Geld zu verdienen.

Akupunktur

Akupunktur gehört zur Traditionellen Chinesischen Medizin. Sie soll gegen die Symptome mancher Erkrankungen helfen und die Selbstheilungskräfte aktivieren. Sie ist dafür bekannt, dass sie den Spiegel der natürlichen Schmerzmittel des Körpers erhöht, und es wurde gezeigt, dass sie Menschen mit chronischen Schmerzen helfen kann. Feine Nadeln werden durch die Haut eingeführt. Die Anzahl der Nadeln variiert, es können bis zu 20 Nadeln, aber auch nur zwei oder drei sein. Im Durchschnitt umfasst die Behandlung ungefähr sechs bis zehn Sitzungen.

Es gibt viele Ärzte in Deutschland, die Akupunktur ausüben dürfen. Es gibt Forschungsergebnisse, die zeigen, dass Akupunktur hilfreich sein kann, wobei das auf einem Placebo-Effekt zu beruhen scheint.

Reflexzonenmassage

Die Reflexzonenmassage ist eine Behandlung, bei der auf eine bestimmte Art und Weise Druck an den Händen oder Füßen ausgeübt wird. Sie soll wegen der Reflexe oder Zonen, die sich durch den Körper ziehen und an den Händen oder Füßen enden, funktionieren. Alle Systeme und Organe des Körpers sollen von einer Region auf der Hand- oder Fußoberfläche repräsentiert werden. Der Therapeut übt sanft Druck auf die passende Region am Fuß oder der Hand aus, um jedwedes Problem in diesem Körperbereich zu behandeln.
Jeder kann lernen, wie man Reflexzonenmassage macht und Reflexzonen-Masseur werden. Es gibt viele Kurse in Deutschland. In Indien und China wird die Reflexzonenmassage seit tausenden von Jahren als Therapie angewandt. Es gibt Forschungsergebnisse, die zeigen, dass sie helfen kann Schmerzen zu reduzieren und sich tief zu entspannen, aber es gibt keine spezifischen Studien, die den Nutzen der Reflexzonenmassage für Menschen mit einem RDS untersuchen. Auch hier könnten Placebo-Effekte eine wichtige Rolle spielen. Reflexzonenmassagen können Menschen helfen, die Reizdarmsymptome haben, die sie mit Stress in Verbindung bringen. Allerdings sind Sie als Patient bei diesem Verfahren sehr passiv. Forschungsergebnisse zeigen, dass bei Krankheiten wie einem RDS Behandlungsmethoden besser helfen, bei denen der Patient selbst mitwirkt und aktiv wird.

Darmspülungen

Bei Darmspülungen wird ein Schlauch ins Rektum eingeführt und eine große Menge Wasser in den Darm geleitet. Darmspülungen werden von Ärzten nicht empfohlen, weil keine gesundheitlichen Vorteile bekannt sind, und sie sind mit ernstzunehmenden Gefahren verbunden, da sie zur Schädigung und zum Einreißen des Dickdarms führen können.

Yoga

Yoga-Übungen sollen den Muskeltonus, die Dehnbarkeit und die Durchblutung verbessern, sowie das geistige Wohlbefinden steigern. Yoga kommt aus einer fernöstlichen Tradition, in der Gesundheit als ein Zustand der Balance zwischen Körper, Verstand und Geist angesehen wird. Es hat sich gezeigt, dass tägliche Yoga-Übungen körperliche Verspannungen reduzieren. Forschungsergebnisse zeigen, dass Yoga helfen kann, sich schneller von mit Stress verbundenen Situationen zu

erholen, Schlaflosigkeit zu verbessern, und Konzentration und Energie zu steigern.

In Yoga-Kursen lernt man eine Körperentspannungstechnik gefolgt von Meditation. Das ist eine Methode, um den Geist zu beruhigen, und den täglichen Stress auszublenden. Durch regelmäßige Meditation kann man lernen sich zu entspannen, wann immer man das Bedürfnis danach hat.

Es gibt auch weitere Techniken, die den ganzen Körper mit einbeziehen und helfen, sich zu entspannen. Beispiele sind autogenes Training, Tai-chi und Qigong. Bei all diesen Techniken sind Sie selbst aktiv. Das kann zu dem positiven Effekt beitragen.

Transzendentale Meditation

Transzendentale Meditation ist eine einfache Art und Weise, sich tief zu entspannen. Sie folgt einer sehr alten Tradition, die vor tausenden von Jahren in Indien begann und jetzt von Millionen von Menschen auf der ganzen Welt praktiziert wird. Man soll sie 20 Minuten morgens und abends ausüben und dabei ein besonderes Wort, „Mantra“ genannt, verwenden. Viele Ärzte empfehlen das ihren Patienten. Die Forschung zeigt, dass transzendentale Meditation den Blutdruck und bestimmte mit Stress verbundene chemische Stoffe im Körper senken kann.

> ***„Ich denke, wenn man sich die Kosten der Pillen anschaut, die angepriesen werden, wäre es besser, das für transzendentale Meditation auszugeben. Weil sie nur 20 Minuten dauert, mache ich das einmal morgens und einmal abends, und es bringt mir wirklich viel.“***

Sie müssen einen Kurs, bei dem Sie die Technik der transzendentalen Meditation lernen, selbst bezahlen.

> ***„Ich habe visuelle Meditation gemacht. Ich versuche mich zu beruhigen, und dann versuche ich, mir bildlich die Tablettenpackung vorzustellen, die, die mich heilen wird. Und es gibt sie in meiner Vorstellung, das Reizdarmsyndrom verliert, es verschwindet. Also weg damit.“***

Mit anderen darüber reden

Man kann sich sehr alleine fühlen, wenn man ein RDS hat. Es hilft, wenn man jemanden hat, der verständnisvoll ist und der einen dabei unterstützt, den besten Weg zu finden, mit dem RDS umzugehen. Vielen kann es helfen mit vom RDS betroffenen Freunden, Kollegen oder Verwandten zu sprechen. Wenn Sie das Gefühl haben, dass Sie lieber mit einem Arzt oder Psychologen über Ihre Probleme sprechen wollen, sprechen Sie Ihren Hausarzt darauf an. Allgemeinmediziner lernen in ihrer Ausbildung auch Grundlagen der psychologischen Beratung, und manche haben sogar eine spezielle Weiterbildung dafür gemacht. Falls Ihr Hausarzt Ihnen nicht persönlich weiterhelfen kann oder Ihre Probleme schwerwiegender sind, kann er Ihnen auch einen Psychotherapeuten empfehlen. Es gibt wissenschaftliche Hinweise, dass psychotherapeutische Verfahren, zum Beispiel die darmbezogene Hypnose, die kognitive Verhaltenstherapie und die psychodynamische Therapie, beim RDS effektiv sind. Mehr dazu finden Sie in Kapitel 5.

> ***„Es hilft unglaublich, wenn man jemanden findet, der zuhört. Manchmal macht es einen riesigen Unterschied, wenn sich wirklich jemand angehört hat, worüber man so nachdenkt.“***

Selbsthilfegruppen können viel Unterstützung bieten. Es gibt viele lokale Selbsthilfegruppen. Sie können zum Beispiel im Internet nach einer Gruppe in Ihrer Nähe suchen.

Pflanzenheilkunde und Reformhäuser

Chinesische Pflanzenheilkunde wird in China seit Jahrhunderten angewandt, um Störungen des Verdauungstrakts zu behandeln. Die Kräutertherapie wird von jemandem, der chinesische Medizin praktiziert, auf die einzelne Person zugeschnitten. Chinesische pflanzliche Heilmittel können schwere Nieren- und Leberprobleme auslösen, wenn sie nicht richtig verwendet werden. Zudem gibt es nicht genug gute Forschungsdaten, um Pflanzenheilkunde als Routinebehandlung

für das RDS zu empfehlen. Aus wissenschaftlicher Sicht wird daher von der Kräutertherapie eher abgeraten.

Viele Menschen mit einem RDS kaufen Mittel im Reformhaus. Es gibt viele verschiedene Produkte, die Sie ausprobieren können, zum Beispiel:
- Pfefferminzöl
- probiotische Produkte, die lebende Bakterien enthalten
- Soja-Milch
- Kräuter-Tees
- Kleie (Weizen- und Haferkleie)

Aloe Vera gibt es in vielen Formen zu kaufen. Allerdings hat sich gezeigt, dass die Wirkung von Aloe Vera nur auf dem Placebo-Effekt beruht und dass sie Nebenwirkungen hat. Daher sollte sie beim RDS eher nicht angewendet werden.

Menschen mit einem RDS haben sehr unterschiedliche Geschichten dazu, was passiert, wenn sie Behandlungen wie Aloe Vera oder pflanzliche Mittel anwenden.

> ***„Ich habe Aloe Vera bestellt, weil es einen Zeitungsartikel dazu gab, wie gut es bei Magenbeschwerden ist, und ich dachte das war's, das würde mich heilen. Es hat absolut nichts gebracht. Aus dem Fenster geschmissenes Geld."***

> ***„Ich habe Pfefferminztee getrunken und das schien die Schmerzen ein bisschen zu beruhigen."***

Ingwer zu kauen soll bei der Verdauung helfen. Kräutertees wie Kamille, Ingwer und Pfefferminze helfen auch.

> ***„Ich habe einfach was ausprobiert, das vor zwei Jahren vorgeschlagen wurde, und es ist wirklich sehr hilfreich, und das ist Rotulme. Man kann sie in Tablettenform kaufen und man kann sie wie eine Paste mischen, das ist was sehr altmodisches, aber abends dieses Pulver und es scheint den Verdauungstrakt zu beruhigen, den ganzen, es hilft gegen die Blähungen und all das."***

Rezeptfreie Behandlungen

Es ist möglich, Medikamente zur Behandlung des RDS rezeptfrei in der Apotheke zu kaufen.
Menschen, die schon lange ein RDS haben, haben manchmal das Gefühl, dass es keine Heilung gibt, die die Ärzte ihnen bieten können. Ärzte neigen dazu, die Symptome des RDS zu behandeln, weil es Medikamente gegen Schmerzen, Verstopfung, Durchfall und Blähungen gibt. In den vielen Fällen können Sie ein Medikament kaufen, das gegen Ihre Symptome hilft. Weil es so viele Reizdarmsymptome gibt, gibt es viele unterschiedliche Behandlungen und was bei dem einen funktioniert, hilft dem anderen vielleicht nicht.

> ***„Ich glaube, dass viele Medikamente gar nicht funktionieren. Man kontrolliert nur den Durchfall, das Reizdarmproblem verschwindet nicht.“***

> ***„Pfefferminzöl-Kapseln sind toll. Die Blähungen, ich hab geknattert wie ein altes Moped, es sind schreckliche Geräusche, aber es hilft.“***

Ein paar Fakten über Medikamente und Nahrungsergänzungsmittel, damit Sie das Richtige für Ihre Symptome auswählen können

Medikamente gegen Verstopfung

Nehmen Sie Abführmittel (Laxantien) nicht regelmäßig ein. Die einfachste Behandlung der Verstopfung ist:

- mehr Ballaststoffe zu essen
- mehr Flüssigkeit zu trinken
- sich mehr zu bewegen. Bewegung hilft gegen Verstopfung, weil es dabei hilft, den Darminhalt weiterzubewegen, wenn man die großen Muskeln des Körpers benutzt. Wenn Sie körperlich nicht aktiv sind, ist es viel wahrscheinlicher, dass Sie Verstopfung bekommen.

Wenn diese Schritte nicht funktionieren, gibt es verschiedene Arten von Abführmitteln, die Sie bei einem RDS kaufen können. Sie haben unterschiedliche Wirkungsweisen im Darm.

Füll- und Quellmittel

Füll- und Quellmittel werden aus pflanzlichen Fasern oder Methylcellulose gewonnen. Sie erhöhen das Stuhlvolumen, indem sie Wasser aufnehmen, was den Stuhl auch weicher macht. Ein großes Stuhlvolumen löst die peristaltischen Bewegungen (Muskelbewegungen der Darmwand) aus. Es kann einige Tage dauern, bevor ein Effekt spürbar ist. Es ist wichtig viel zu trinken, wenn man Quellmittel einnimmt, um Darmblockaden zu vermeiden.
Sie sollten Füll- und Quellmittel morgens oder abends mit einem Glas Wasser einnehmen – nicht direkt vor dem Zubettgehen.

Beispiel für Füll- und Quellmittel:
- Indische Flohsamen (Psyllium) – z.B. Agiocur®, Flosine®, Metamucil®, Mucofalk®, Pascomucil®.

Osmotische Laxantien

Osmotische Laxantien ziehen Wasser in das Darminnere und sorgen so dafür, dass der Stuhl leichter passieren kann. Es ist wichtig viel zu trinken, wenn Sie diese Abführmittel einnehmen.

Beispiel für osmotische Laxantien:
- Macrogol – z.B. Isomol®, Laxofalk®, Movicol®.
- Lactulose – z.B. Bifiteral®, Lactuflor®, LactuGel®, Lactulade® – sollten Sie beim RDS eher nicht einnehmen, da Blähungen eine häufige Nebenwirkung sind.

Stimulierende Laxantien

Menschen mit einem RDS brauchen keine stimulierenden Abführmittel einzunehmen. Sie verstärken die Muskelbewegungen im Dickdarm und können schmerzhafte Krämpfe verursachen. Es dauert ungefähr 12 Stunden, bis sie wirken.

Beispiel für stimulierende Laxantien:
- Senna – z.B. Alasenn®, Bekunis®, Midro®, Neda®, Ramend®.
- Bisacodyl - z.B. Dulcolax®

Medikamente gegen Durchfall

Es gibt Medikamente, die helfen, die Muskelbewegungen im Darm zu verlangsamen. Diese Medikamente heißen Motilitätshemmer.

Beispiel für Motilitätshemmer:
- Loperamid – z.B. Imodium®, Loperhoe®.

Wenn Sie manchmal Verstopfung und manchmal Durchfall haben, können Abführmittel und Medikamente gegen Durchfall die Symptome verschlimmern.

Medikamente gegen Schmerzen

Es gibt Medikamente, die die Darmmuskulatur entspannen. Man denkt, dass Muskelkrämpfe die Ursache der Schmerzen sind, unter denen mache Menschen mit einem RDS leiden.
Es gibt krampflösende Medikamente zu kaufen, die die Darmmuskulatur entspannen.

Beispiel für krampflösende Medikamente:
- Pfefferminzöl – z.B. spasmo gallo sanol®.

Anticholinergika

wirken auf die Nervenversorgung der Darmmuskulatur.

Beispiel für Anticholinergika:
- Butylscopolamin – Buscopan®. Buscopan kann bei Patienten mit einem RDS gelegentlich Nebenwirkungen wie Durchfall, Müdigkeit und Magenbeschwerden verursachen.

Blähungen

Es gibt pflanzliche Präparate, die bei Blähungen helfen können.

Beispiel für pflanzliche Präparate:
- STW 5 – z.B. Iberogast®.

Beispiele für weitere Medikamente gegen Blähungen:
- Aktivkohle
- Simeticon – z.B. Elugan®, Espumisan®, Lefax®, sab simplex®.
- Dimeticon – z.B. Ceolat®, ILIO-FUNKTON®, sab simplex®.

„Kohletabletten haben mir wirklich bei diesem peinlichen Durchfall-Problem geholfen."

Denken Sie daran …

… Komplementäre Therapien können Menschen mit einem RDS helfen, sollten aber nicht zu teuer sein.

… Mittel gegen das RDS finden Sie zum Beispiel in der Apotheke oder im Reformhaus.

… Rezeptfreie Medikamente können gegen Symptome des RDS helfen.

Kapitel 5

Ärztliche Behandlung

In diesem Kapitel geht es um die ärztliche Betreuung und Therapien, mit denen man Reizdarmsymptome behandeln kann.

Was können Sie tun, wenn nichts zu helfen scheint?

In diesem Kapitel geht es um die medizinischen Behandlungen, mit denen man Menschen mit einem RDS hilft, die die Krankheit alleine nicht bewältigen können. Ungefähr 95% der Menschen mit einem RDS sollten es schaffen, selbst mit ihrem RDS fertig zu werden, indem sie die einfachen Methoden in diesem Ratgeber anwenden. Aber es gibt eine kleine Gruppe von Menschen, die sehr starke Symptome haben, denen durch keine Behandlung geholfen ist. Falls keine der Methoden aus den Kapiteln 3 oder 4 in diesem Ratgeber geholfen haben, kann es sein, dass Sie über andere Therapien nachdenken müssen.

Verschreibungspflichtige Medikamente

Es gibt ein paar Behandlungsformen, die rezeptpflichtig sind. Diese können Sie nur von Ihrem Arzt verordnet bekommen. Die folgenden Medikamente sind auf Rezept erhältlich und weitere Informationen können Sie im Patienteninfo-Service der Rote Liste GmbH finden. Die Rote Liste ist ein Handbuch, in dem alle rezeptpflichtigen Medikamente aufgelistet sind.

Medikamente gegen Verstopfung

Prokinetika verstärken die Bewegungen der Darmmuskulatur und können deshalb dafür sorgen, dass der Stuhl besser transportiert wird, und somit gegen Verstopfung helfen.

Beispiel für Prokinetika:
- Prucaloprid – Markenname: Resolor®

Medikamente, die die Sekretion (Ausschüttung von Wasser in den Darm) fördern, können ebenfalls bei Verstopfung wirken. Sie heißen Sekretagoga.

Beispiel für Sekretagoga:
- Linaclotid – Markenname: Constella®

Therapie mit Antidepressiva

Forschungsergebnisse zeigen, dass niedrige Dosen von Medikamenten gegen Depressionen gegen manche Reizdarmsymptome helfen können. Ein RDS kann auch ein Anzeichen einer Depression sein. Man merkt vielleicht gar nicht, dass man depressiv ist. Depressionen wirken sich auf den Körper aus, wodurch man Schmerzen leichter wahrnimmt, die Krämpfe und Bewegungen der Darmmuskulatur werden als schmerzhafter empfunden. Menschen mit Depressionen fühlen sich müde und wollen sich nicht bewegen, und Bewegungsmangel verursacht Verstopfung. Antidepressiva können sowohl helfen, mit der Depression umzugehen, als auch die Schmerzsymptome zu lindern. Ihr Hausarzt sollte Sie überwachen, wenn Sie Antidepressiva nehmen, und Ihre komplette Medikamentenliste überprüfen, weil man Antidepressiva nicht mit manchen anderen Medikamenten zusammen einnehmen sollte, weil dies sonst gefährliche Nebenwirkungen haben kann.

Trizyklische Antidepressiva

Es wurde gezeigt, dass trizyklische Antidepressiva auf die Darmmuskulatur wirken und manchen Menschen, die Bauchschmerzen haben, helfen können. Diese Medikamente haben manchmal Nebenwirkungen, wie zum Beispiel Müdigkeit und Mundtrockenheit. Diese Nebenwirkungen lassen in der Regel nach ein paar Tagen nach.
Trizyklische Antidepressiva können Sie bei Durchfall und Bauchschmerzen einnehmen, Sie sollten sie aber meiden, wenn Sie unter Verstopfung leiden, weil sie diese verstärken können.

Beispiele für trizyklische Antidepressiva, die beim RDS angewendet werden:
- Amitriptylin – z.B. Saroten®, Syneudon®
- Imipramin – z.B. Tofranil®

Serotonin-Wiederaufnahmehemmer (SSRI)

SSRI sind eine andere Art von Antidepressiva, die bei einem RDS helfen können. SSRI können Sie bei Verstopfung einnehmen, Sie sollten sie bei Blähungen aber meiden.

Beispiel für SSRI:
- Fluoxetin – z.B. Fluxet®

Kognitive Verhaltenstherapie (KVT)

Es gibt Hinweise darauf, dass die KVT bei einem RDS helfen kann. Mit der KVT kann Ihnen geholfen werden, die Veränderungen herauszufinden, die das RDS in Ihrem Leben verursacht hat. Durch die KVT lernen Sie besser zu verstehen, wie Denken, Handeln und Gefühle miteinander verbunden sind. Das hilft Ihnen, andere Strategien zu planen, wie Sie besser mit Ihren Reizdarmsymptomen umgehen können.
Die KVT ist relativ kurz und umfasst regelmäßige Termine über mehrere Monate. Sie und Ihr Therapeut werden zusammenarbeiten, um Ihre Probleme zu verstehen und gemeinsam einen Plan zu erstellen, wie Sie mit Ihren Beschwerden umgehen können. Sie werden einige Dinge lernen, die Sie tun können, um sich selbst zu helfen, unter anderem vielleicht:

- wie Sie Ihre Aktivitäten einteilen können
- wie Sie sich Ziele setzten können, um Ihr Verhalten zu ändern
- ein Tagebuch zu führen
- nicht hilfreiche oder negative Denkmuster, in die Sie hineingeraten sind, zu identifizieren und mit ihnen umzugehen
- einen Langzeitplan zum Umgang mit dem RDS zu erstellen

Psychotherapie

Psychotherapie ist eine Behandlung, die Ihnen hilft, die körperlichen Auswirkungen, die emotionale Belastungen und Stressfaktoren auf Sie haben, besser zu verstehen. Das könnten Sorgen über Geld, Ihre Familie oder Ihren Beruf sein. Es ist bekannt, dass psychische Belastungen Darmprobleme hervorrufen können. Es wurde gezeigt, dass Ihre Darmsymptome sich verbessern können, wenn Ihnen bei Angsterkrankungen und Depressionen geholfen wird. Für die Psychotherapie ist ein gutes Verhältnis zu Ihrem Therapeuten wichtig. Wenn

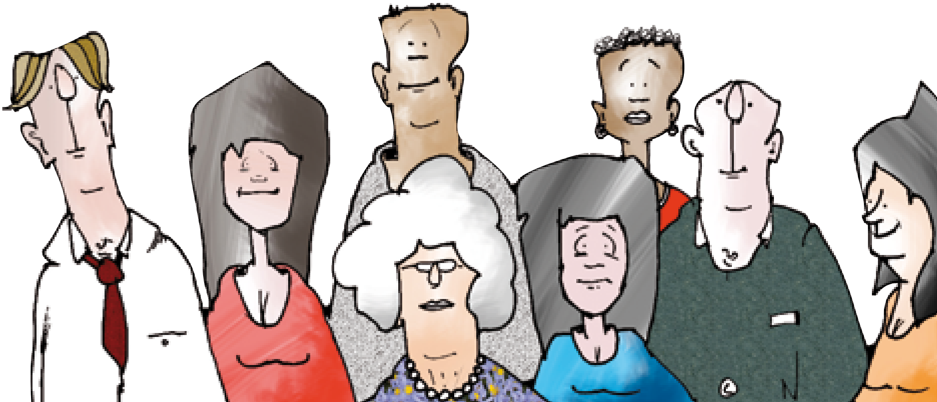

Sie das Gefühl haben, dass Sie einen Psychotherapeuten aufsuchen sollten, kann Ihr Hausarzt Sie überweisen, oder Sie können selbstständig einen Psychotherapeuten kontaktieren.

Hypnotherapie

Hypnose ist ein Weg, einen Zugang zum Unterbewusstsein zu bekommen. Mit Hilfe der Selbsthypnose können Sie lernen, ihr Bewusstsein anders zu benutzen. Eine Trance unter Hypnose ist ein Zustand fokussierter Aufmerksamkeit. Dies ist in etwa vergleichbar damit, dass Sie so von einem Buch oder Film gefangen genommen sind, dass Sie nicht hören, was jemand zu Ihnen sagt.
Hypnotherapeuten können unterschiedliche Therapieformen zur Therapie des RDS anwenden. Es wird nicht streng kontrolliert, wer Hypnotherapie durchführen darf. Wenn Sie sich entscheiden, Hypnotherapie auszuprobieren, ist es wichtig, dass Sie zu jemandem gehen, der Erfahrung mit darmbezogener Hypnotherapie hat. Gehen Sie zu einem zertifizierten Therapeuten, der Ihnen von jemandem empfohlen wurde, dem Sie vertrauen.
Hypnotherapie kann angewendet werden, um Ihnen zu helfen sich zu entspannen und Stress zu überwinden. Allerdings profitiert nicht jeder von Hypnotherapie.

Darmbezogene Hypnotherapie

Bei darmbezogener Hypnotherapie wird die Behandlung gezielt auf die Reizdarmprobleme ausgerichtet. Es wurde gezeigt, dass diese Methode bei manchen Patienten mit einem starken RDS funktioniert. Der Darm kann vom Geist beeinflusst werden, daher sagen Hypnotherapeuten, dass sie ein Handwerkszeug vermitteln, wie Sie Ihren Darm mit dem Willen kontrollieren können. Es ist kein Heilmittel, aber viele Menschen, berichten von einer Besserung ihrer Symptome. Sie fühlen sich wohler mit sich selbst und besser fähig, mit dem Leben zurechtzukommen.

Hier nur ein Beispiel für darmbezogene Hypnotherapie

- Es wird Ihnen erklärt, wie der Darm funktioniert und was damit nicht stimmen könnte.
- Sie bekommen drei Monate lang einmal pro Woche eine einstündige Hypnotherapie-Sitzung.
- Die Hypnotherapie ist auf den Darm gerichtet. Sie werden angewiesen, die Hand auf den Bauch zu legen und die Wärme zu spüren.

Dann wird Ihnen gesagt, dass Sie dieses Gefühl mit der Linderung von Schmerz, Krämpfen und Blähungen durch das RDS verbinden sollen. Sie werden auch gebeten, sich ein Bild eines Flusses zu überlegen und sich vorzustellen, dass er Ihr Darm ist. Ihnen wird gesagt, Sie sollen die Art wie der Fluss fließt so verändern, dass Ihr Darm besser funktioniert. Wenn Sie zum Beispiel Probleme mit weichen Stühlen haben, werden Sie gebeten sich vorzustellen, dass ein schnell fließender Fluss in einen ruhig und langsam fließenden verwandelt wird.

- Sie werden gebeten, zu Hause Selbsthypnose mit einem Tonband zu machen.

Operationen und das RDS

Es gibt aussagekräftige Daten dafür, dass Patienten mit einem RDS mehr unnötige Operationen wegen ihrer Schmerzen haben, zum Beispiel gynäkologische Operationen, Gallenblasenoperationen und Blinddarmentfernungen. Diese Operationen helfen nicht gegen die Schmerzen und können die Lage manchmal verschlimmern. Sie sollten sich wegen eines RDS nicht operieren lassen.

Wenn Sie aus anderen Gründen operiert werden müssen, sollten Sie versuchen, starke Schmerzmittel zu vermeiden, die auf Opiaten basieren, zum Beispiel Morphin, weil sie die Darmfunktion und die Schmerzen verschlimmern können.

Denken Sie daran …

… Manche Menschen mit einem RDS können von medizinischer Hilfe profitieren.

… Verschreibungspflichtige Medikamente können vermehrt Nebenwirkungen haben.

… Nur wenige Menschen brauchen professionelle psychologische Hilfe.

Kapitel 6

Zusammenfassung und Informationsquellen

Dieses Kapitel fasst noch einmal zusammen, was Sie tun können, um mit Ihrem RDS zurechtzukommen und es zu handhaben.

Denken Sie daran, dass es kein Allheilmittel für das RDS gibt. Wir wissen aber, dass Sie Ihre Lage verbessern können, wenn Sie die Kontrolle übernehmen und Ihr RDS steuern, anstatt sich vom RDS steuern zu lassen.

„Ich glaube, es gibt etwas für jeden, das hilft, besser damit umzugehen. Es geht nur darum, das Richtige für die jeweilige Person zu finden, oder?"

Bewältigungsstrategien

1. **Seien Sie sich im Klaren darüber, was Ihre Symptome sind und welche Symptome Sie am meisten stören. Überprüfen Sie, ob Sie die Symptome haben, die am Ende von Kapitel 2 aufgelistet sind.**
2. **Sie haben keine gefährliche Krankheit. Machen Sie sich nicht zu viele Sorgen.**
3. **Versuchen Sie herauszufinden, was Ihr RDS auslöst.**
4. **Ändern Sie Ihre Ernährung, wenn das nötig ist.**
5. **Denken Sie darüber nach, was bei Ihnen Stressgefühle auslöst.**
6. **Bewegen Sie sich mehr.**
7. **Es kann helfen, wenn man lernt, wie man sich richtig entspannt.**
8. **Es kann helfen, jemand anderen mit einem RDS zu finden und mit ihm darüber zu reden.**
9. **Überlegen Sie sich, ob komplementäre Therapien Ihnen helfen könnten.**
10. **Arbeiten Sie mit Ihrem Arzt oder Therapeuten zusammen, um eine Methode zu finden, Ihre Symptome zu lindern.**

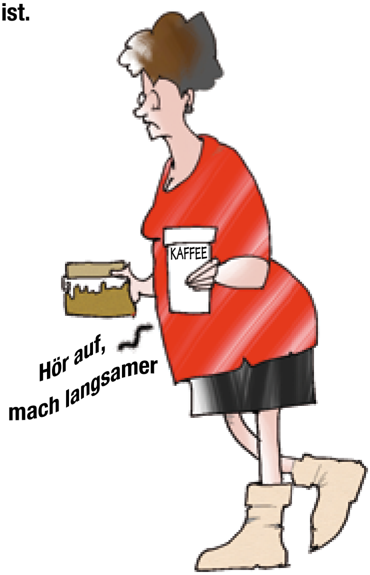

Allgemeine Tipps

1. Wenn Sie sich nicht schon ballaststoffreich ernähren, erhöhen Sie langsam die Ballaststoffmenge, die Sie zu sich nehmen. Die meisten Menschen brauchen mehr Ballaststoffe. Ballaststoffe helfen gegen Verstopfung und können bei Durchfall helfen. Wenn Sie plötzlich anfangen, große Mengen an ballaststoffhaltigen Nahrungsmitteln zu sich zu nehmen, können Sie Bauchschmerzen und Krämpfe bekommen, weil Ihr Körper versucht, sich an die neue Ernährung zu gewöhnen. Sie werden auch stärkere Blähungen haben, bis Ihr Körper lernt, mit den zusätzlichen Ballaststoffen zurechtzukommen. Das kann zwei bis drei Wochen dauern. Manche Menschen mit einem RDS können mit einer ballaststoffreichen Ernährung nicht umgehen, weil sie ihre Symptome verschlimmert. Sie können das nur durch Erfahrung herausfinden.
2. Trinken Sie mehr Wasser. Wasser hilft gegen Verstopfung und Durchfall. Sie sollten versuchen, mindestens zwei Liter am Tag zu trinken.
3. Entwickeln Sie gute Stuhlgewohnheiten.
   - Nehmen Sie nicht regelmäßig Abführmittel ein.
   - Wenn Sie das Bedürfnis haben den Darm zu entleeren, versuchen Sie nicht, es zu unterdrücken, versuchen Sie, so bald wie möglich zu gehen.
   - Strengen Sie sich nicht zu sehr an, wenn Sie versuchen, Stuhlgang zu haben. Wenn es nicht ungefähr innerhalb einer Minute klappt, stehen Sie von der Toilette auf und warten Sie, bis der Drang wiederkommt.
   - Versuchen Sie nicht, Blähungen zu unterdrücken. Lassen Sie die Winde ab.
4. Bewegen Sie sich öfter. Wenn Sie Ihre Bauchmuskeln benutzen, hilft das, den Darminhalt normaler fortzubewegen.
5. Probieren Sie einfache Methoden der Schmerzlinderung aus – eine Wärmflasche auf dem Bauch sollte beruhigend wirken.

„Ich lege mich in die Badewanne und lasse immer wieder heißes Wasser nachlaufen.“

„Ich lege mich mit einer Wärmflasche ins Bett und bleibe da.“

„Ich habe das Gefühl, dass es mir hilft, mich hinzusetzen und mir ein bisschen mehr Zeit zu nehmen, bevor ich wieder durch die Gegend renne.“

Detektivarbeit

Sie müssen herausfinden, was mit Ihrem Körper passiert. Denken Sie über Ihre Symptome nach.

- Welche Symptome haben Sie?
- Was können Sie dagegen tun?
- Können Sie die Symptome mit irgendeinem Ereignis in Ihrem Leben in Verbindung bringen?
- Könnte Ihr RDS mit Nahrungsmitteln zusammenhängen?

Handeln

Wenn Sie das Gefühl haben, dass Sie Ihr RDS nicht unter Kontrolle haben, kann es helfen etwa zwei Wochen lang ein Tagebuch über Ihre Symptome zu führen. Sie können festhalten wann die Attacken kommen, wie schlimm sie sind und ob Sie mit irgendetwas zusammenhängen, zum Beispiel mit dem was Sie gegessen haben, mit Problemen in der Arbeit oder Problemen zu Hause.

Medikamente

Finden Sie heraus, welche Medikamente bei Ihren Symptomen helfen. Versuchen Sie, ein Medikament zu finden, das die Symptome bekämpft, unter denen Sie am meisten leiden. Es gibt kein einzelnes Medikament, das jedem mit einem RDS hilft.

Probleme beim Sex

Viele Frauen und Männer mit starken Reizdarmsymptomen sagen, dass sie Probleme beim Sex haben. Das Hauptproblem, das sie haben, ist ein verminderter Geschlechtstrieb. Bei Menschen, die ein RDS mit Verstopfung haben, sind sexuelle Probleme wahrscheinlicher. Es muss Ihnen klar sein, dass der Verlust des Geschlechtstriebs ein häufiges Problem bei Menschen mit chronischen oder psychosomatischen Beschwerden ist. Wenn für Sie Probleme beim Sex ein großes Anliegen sind, kann Ihnen eine Beratung helfen. Ihr Hausarzt kann Ihnen helfen, das in die Wege zu leiten.

Informationen und das Internet

Es gibt viele Informationen zum RDS im Internet. Viele Menschen mit einem RDS benutzen das Internet, um zu kommunizieren und Ideen und Erfahrungen auszutauschen. Es gibt drei Haupttypen von Webseiten, die Informationen für Menschen mit einem RDS enthalten.

1. Medizinische Webseiten

Diese Seiten werden von Krankenhäusern oder gemeinnützigen medizinischen Einrichtungen betrieben und enthalten verlässliche Informationen über die Krankheit. Sie sind für Menschen nützlich, die mehr Informationen über ihre Krankheiten haben möchten, oder um etwas über die aktuelle Forschung zu Aspekten der Krankheit herauszufinden.

2. Persönliche Webseiten

Diese Webseiten werden normalerweise von Menschen mit einem RDS geschrieben, die ihre Erfahrungen teilen oder mit anderen Internetbenutzern kommunizieren wollen. Jeder kann seine eigene Webseite einrichten. Die meisten persönlichen Webseiten sind ehrliche Versuche von Menschen mit einem RDS, anderen zu helfen, indem sie Ratschläge geben und sie wissen lassen, dass sie nicht alleine leiden. Diese Webseiten werden manchmal auch als Pinnwand genutzt, auf der man Nachrichten für andere Leser hinterlassen kann.
Es gibt keine zwei Patienten, die exakt die gleichen Symptome oder Krankheitsmuster haben, und manche Erfahrungen oder Behandlungen, die von Patienten beschrieben werden, sind für Sie vielleicht nicht relevant.

3. Werbung

Die meisten Webseiten, die auf Menschen mit einem RDS ausgerichtet sind, versuchen, Ihnen etwas zu verkaufen. Manche dieser Seiten sind offensichtlich Werbung, aber viele erscheinen als persönliche Seiten, auf denen der Autor behauptet, dass ein bestimmtes Produkt bei seiner Krankheit geholfen hätte. Sie sollten diesen Behauptungen gegenüber misstrauisch sein, dass ein Produkt geholfen oder die Person geheilt hätte, da es vielleicht nicht richtig getestet wurde und das der Grund ist, aus dem es im Internet angeboten wird. Menschen mit chronischen Krankheiten sind oft verletzlich und sind bereit, alles auszuprobieren, das gegen ihre Symptome hilft. Wenn Sie von einem Produkt hören, von dem Sie denken, dass es hilfreich sein könnte, versuchen Sie mehr darüber herauszufinden. Eine Bibliothek oder ein Apotheker hat vielleicht mehr Informationen. Aber bitte sehen Sie die Behauptungen oder Empfehlungen der Menschen nicht einfach als Fakten an.

Notieren Sie Ihre Lieblingswebseiten hier:

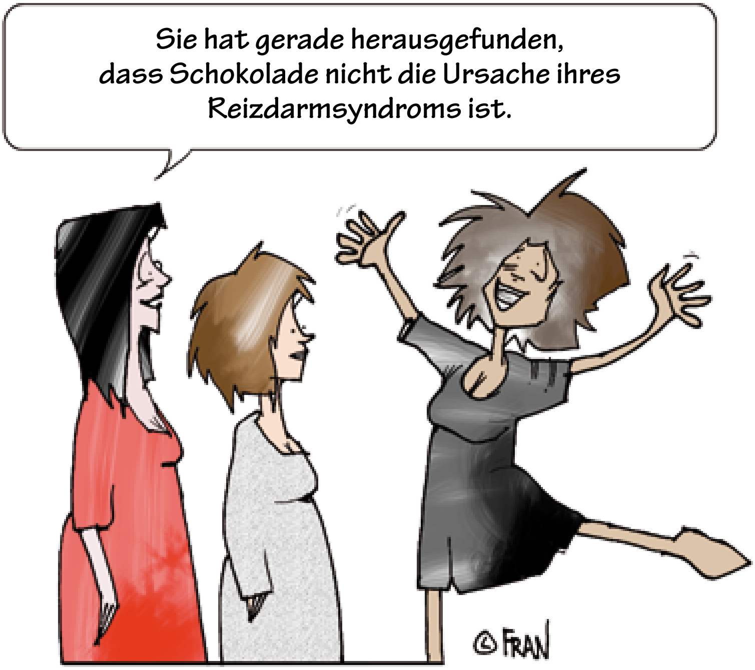

Aktuelle Forschung zum RDS

In den letzten paar Jahren wurde viel am RDS geforscht. Die Forschung bewegt sich in zwei Hauptgebieten:
- herauszufinden, welchen Einfluss das RDS auf das Leben der Menschen hat und
- neue Behandlungsmöglichkeiten zu finden.

Dieser Ratgeber war Teil einer erfolgreichen Studie, die betrachtet hat, wie Menschen ihr RDS selbst steuern können.
Viele der Studien haben mögliche medikamentöse Behandlungsweisen des RDS untersucht. Diese Studien zeigen, dass bestimmte neue Medikamente bei verschiedenen Reizdarmsymptomen helfen können. Es werden neue Medikamente entwickelt, die die Schmerzen kontrollieren sollen, unter denen Menschen mit einem RDS leiden. Andere Studien untersuchen Hormone und andere chemische Stoffe, die an der Darmfunktion beteiligt sind. Manche chemischen Stoffe sind dafür bekannt, dass sie die Muskelaktivität im Dickdarm reduzieren, und andere machen den Dickdarm weniger empfindlich. Bisher hat die Forschung gezeigt, dass sie sich bei Menschen mit einem RDS nicht als effektiv erwiesen haben, obwohl diese chemischen Stoffe im Zusammenhang mit Reizdarmsymptomen relevant sein könnten. Es gibt keine Hinweise darauf, dass man ein Medikament finden wird, das alle Reizdarmsymptome heilt.

Den Forschern stehen immer mehr neue Techniken zur Verfügung, um Studien durchzuführen, mit denen besser verstanden werden kann, was im Körper von Menschen mit einem RDS passiert. Diese Techniken messen die Muskelbewegungen des Darms und können benutzt werden, um herauszufinden, wie sensibel der Darm auf Schmerz reagiert. Aktuelle Studien helfen dabei zu erklären, welche Teile des Darms sich bei Menschen mit einem RDS am stärksten zusammenziehen. Die Forschung hat gezeigt, dass bei Menschen mit einem RDS bestimmte Teile des Verdauungssystems sehr sensibel auf Druck und Schmerz reagieren. Wenn mehr über die Vorgänge in den verschiedenen Teilen des Darms bekannt ist und man weiß, was bei Menschen mit einem RDS falsch läuft, können Medikamente entwickelt werden, die bei diesen Problemen helfen.

Forscher untersuchen auch, wie sich das RDS auf die Menschen auswirkt. Zum Beispiel:
- ob etwas, das in der Kindheit passiert ist, zu einem RDS führen kann
- Umfragen, wie lange die Symptome dauern und wie oft sie kommen
- Umfragen, wie sehr das RDS sich auf den Alltag auswirkt

Weitere aktuelle Forschung deckt die folgenden Theorien ab:
- ob das RDS mit dem Menstruationszyklus zusammenhängt
- ob das RDS vererbbar ist
- ob die Anatomie der Menschen mit einem RDS anders ist
- ob das RDS mit sexuellem Missbrauch zusammenhängt
- wie das RDS mit Ereignissen zusammenhängt, die mit Stress verbunden sind
- wie das RDS mit der Wahrnehmung des Darms im Gehirn zusammenhängt
- ob es einen Zusammenhang zwischen Essen und Reizdarmsymptomen gibt

Neue Behandlungen, die untersucht werden, sind unter anderem:
- die Anwendung von Probiotika;
- Spasmolytika (krampflösende Medikamente), die gezielt auf den Darm wirken, so dass sie weniger Nebenwirkungen haben;
- Medikamente, die die Darmbewegungen verstärken und deshalb bei Verstopfung helfen können.

Weitere Forschung beschäftigt sich unter anderem mit:
- der Effektivität von Selbstmanagement-Ratgebern;
- epidemiologischen oder Bevölkerungsstudien;
- dem Einfluss des RDS auf den Lebensstil und Beziehungen;
- Studien, die untersuchen, wie man vorhersagen kann, wer ein RDS entwickeln wird.

Diese Forschung hilft den Ärzten, besser zu verstehen, was mit Menschen mit einem RDS passiert, aber bisher kann niemand genau sagen, was das RDS verursacht oder wann es ein Heilmittel für das RDS geben wird.

! Wir wissen aber, dass die Wahrscheinlichkeit größer ist, dass Sie sich besser fühlen, wenn Sie die Kontrolle übernehmen und Ihr RDS steuern, statt sich davon steuern zu lassen.

Ihre Notizen:

Hilfreiche Quellen für weitere Informationen

Das Reizdarmsyndrom. 44 Fragen – 44 Antworten

MÖNNIKES, H.: Das Reizdarmsyndrom. 44 Fragen – 44 Antworten. Thieme Verlag 2014

Gesundheitsinformation.de

http://www.gesundheitsinformation.de

MAGDA

Patientenforum der Deutschen Gesellschaft für Neurogastroenterologie und Motilität e.V. (DGNM)

MAGDA
DGNM-Geschäftsstelle
Gastro-Haus
Olivaer Platz 7
10707 Berlin

http://www.neurogastro.de/magda.html

Glossar medizinischer Fachbegriffe

Abdomen: Bauch
Allergie: Eine Funktionsstörung des Immunsystems, bei der der Körper nicht normal auf einen bestimmten Stoff reagiert. Die Symptome sind immer die gleichen und verschwinden, wenn man den Stoff, der sie verursacht, entfernt.
Anus: Die Körperöffnung, durch die der Stuhl den Körper verlässt.
Biopsie: Eine kleine Gewebeprobe wird während einer Endoskopie und Koloskopie aus der Darmwand entnommen und mit Hilfe eines Mikroskops untersucht.
Chronisch: Lang anhaltend.
Duodenum: Zwölffingerdarm, der erste Teil des Dünndarms. Das Duodenum ist mit dem Magen verbunden.
Endoskop: Ein Instrument, das verwendet wird, um den Körper von innen zu betrachten.
Enzym: Ein Eiweiß, das eine biologische Reaktion beschleunigt. Enzyme sind lebenswichtig dafür, dass der Körper normal funktioniert.
Fäzes: Stuhl
Flatulenz: Blähungen
Fruktose: Fruchtzucker
Gastroenterologe: Ein Arzt, der besonders im Bezug auf das Verdauungssystem ausgebildet ist.
Gastrointestinal: Mit dem Verdauungssystem zusammenhängend.
Hypersensibel: Wenn der Körper auf etwas überreagiert.
Ileum: Der letzte Teil des Dünndarms. Es geht in den Dickdarm über.
Jejunum: Der mittlere Teil des Dünndarms. Es verbindet das Duodenum mit dem Ileum.
Kolon: Dickdarm
Koloskopie: Dickdarmspiegelung. Ein langer biegsamer Schlauch wird durch den Anus eingeführt und sendet Bilder an einen Videoempfänger. Das wird durchgeführt, um den gesamten Dickdarm zu untersuchen.

Laktase: Ein Enzym, das während des Verdauungsprozesses Laktose spaltet, so dass sie in den Körper aufgenommen werden kann.
Laktose: Milchzucker
Laxans: Abführmittel, ein Medikament, das man verwendet um den Darm zu entleeren.
Lebensmittelintoleranz: Wenn ein bestimmtes Nahrungsmittel Beschwerden und Krankheit hervorruft.
Mukus: Ein weißer Schleim, den der Darm produziert, um den Lebensmitteln die Passage zu erleichtern.
Nährstoffe: Lebensmittelbestandteile, die der Körper braucht, um richtig zu wachsen und zu funktionieren.
Ösophagus: Speiseröhre, er verbindet den Mund mit dem Magen.
Präbiotikum: Eine Substanz, die das Bakterienwachstum unterstützt.
Probiotikum: Lebende Bakterien, die verwendet werden, um die Darmbakterien zu ersetzen oder deren Gleichgewicht zu verändern.
Nebenwirkungen: Ungewollte Wirkungen von Medikamenten.
Sigmoidoskopie (flexibel): Ein kurzer biegsamer Schlauch, der durch das Rektum eingeführt wird und Bilder an einen Videoempfänger übermittelt. Damit kann man den letzten Teil des Dickdarms betrachten.
Sigmoidoskopie (starr): Ein kurzer Plastikschlauch, der ein Licht enthält, wird durch den Anus eingeführt, um das Rektum zu betrachten.
Stuhldrang: Das Bedürfnis, sofort auf die Toilette zu gehen und den Darm zu entleeren.

Literaturverzeichnis

Kennedy A: Managing your life with Irritable Bowel Syndrome. 2. Aufl. Manchester : The University of Manchester, 2009

Layer P, Andresen V, Pehl C, Allescher H, Bischoff S, Classen M, et al.: S3-Leitlinie Reizdarmsyndrom – Definition, Pathophysiologie, Diagnostik und Therapie. Gemeinsame Leitlinie der Deutschen Gesellschaft für Verdauungs- und Stoffwechselkrankheiten (DGVS) und der Deutschen Gesellschaft für Neurogastroenterologie und Motilität (DGNM). In : Zeitschrift für Gastroenterologie, 49 (2011), Nr. 2, S. 237–293.

Raithel M, Weidenhiller M, Hagel AF, Hetterich U, Neurath MF, Konturek PC: The malabsorption of commonly occurring mono and disaccharides – levels of investigation and differential diagnoses. In : Dtsch Arztebl Int, 110 (2013), Nr. 46, S. 775–782.

Vielleicht hilft es Ihnen, unterschiedliche Selbsthilfe-Behandlungen auszuprobieren und aufzuschreiben, wie sehr sie Ihnen geholfen haben. Sie können auf diese Notizen zurückkommen, falls das RDS in der Zukunft zu

	THERAPIE	Datum
1	Yoga	
2	Meditation	
3	Bewegung	
4	Einer RDS-Selbsthilfegruppe beigetreten	
5	Hypnotherapie	
6	Andere Therapien	

	MEDIKAMENTE	Name des Medikaments / Datum
7	Gegen Verstopfung	
8	Gegen Durchfall	
9	Gegen Schmerzen	

einem Problem wird. Die Notizen können auch im Gespräch mit Ihrem Arzt helfen, wenn Sie ihn wegen Ihres RDS aufsuchen.

	Hat es geholfen?		Kommentar
1	Ja ☐	Nein ☐	
2	Ja ☐	Nein ☐	
3	Ja ☐	Nein ☐	
4	Ja ☐	Nein ☐	
5	Ja ☐	Nein ☐	
6	Ja ☐	Nein ☐	

	Hat es geholfen?		Kommentar
7	Ja ☐	Nein ☐	
	Ja ☐	Nein ☐	
	Ja ☐	Nein ☐	
	Ja ☐	Nein ☐	
8	Ja ☐	Nein ☐	
	Ja ☐	Nein ☐	
	Ja ☐	Nein ☐	
	Ja ☐	Nein ☐	
9	Ja ☐	Nein ☐	
	Ja ☐	Nein ☐	
	Ja ☐	Nein ☐	
	Ja ☐	Nein ☐	

Ernährungs-Wochenplan

Schreiben Sie für jeden Tag auf, was Sie gegessen haben.

	Montag	Dienstag	Mittwoch
**Frühstück**			
**Vormittag**			
**Mittagessen**			
**Nachmittag**			
**Abendessen**			
**Zwischendurch**			
**Symptome**			
**möglicher Stress**			

Ernährungs-Wochenplan

	Donnerstag	Freitag	Samstag	Sonntag
Frühstück				
Vormittag				
Mittagessen				
Nachmittag				
Abendessen				
Zwischendurch				
Symptome				
mögl. Stress				

Englische Originalversion von:
Anne Kennedy
Dr. Andrew Robinson

Übersetzt und überarbeitet von:
Stefanie Rosenberger
Prof. Dr. med. Antonius Schneider
Prof. Dr. Paul Enck
Dr. med. Jessica Bungartz

Layout:
büro bartl

Klinikum rechts der Isar
der Technischen Universität München
Institut für Allgemeinmedizin
Univ.-Prof. Dr. med. Antonius Schneider
Orleansstr. 47, 81667 München
Germany
