# Supplementary material for: Self-help guidebook improved quality of life for patients with irritable bowel syndrome
Source: PLoS One. 2017 Jul 25;12(7):e0181764. doi: 10.1371/journal.pone.0181764 (PMC5526555; doi:10.1371/journal.pone.0181764)
Supplement: S2 Table — (DOCX) [file pone.0181764.s003.docx]

**Coding of variables**

| **Responder at t2 and t3** |  |
| --- | --- |
| No | 0 |
| Yes | 1 |
|  |  |
| **Sex** |  |
| Female | 1 |
| Male | 2 |
|  |  |
| **Education** |  |
| No school leaving certificate | 1 |
| Special school | 2 |
| Lower secondary school certificate | 3 |
| Secondary school certificate | 4 |
| Educated to degree level | 5 |
| Other | 6 |
|  |  |
| **Depression / anxiety / somatization categorical** |  |
| Present | 1 |
| Not present | 0 |

**All other variables are calculated as means**
